# Tailoring the Morphology of Cellulose Nanocrystals via Controlled Aggregation

**DOI:** 10.1021/acsnano.5c05548

**Published:** 2025-07-02

**Authors:** Kévin Ballu, Jia-Hui Lim, Thomas G. Parton, Richard M. Parker, Bruno Frka-Petesic, Alexei A. Lapkin, Yu Ogawa, Silvia Vignolini

**Affiliations:** † Yusuf Hamied Department of Chemistry, 2152 University of Cambridge, Cambridge CB2 1EW, U.K.; ‡ 55139 University of Grenoble Alpes, CNRS, CERMAV, Grenoble 38000, France; § Department of Sustainable and Bio-inspired Materials, Max Planck Institute of Colloids and Interfaces, Potsdam 14476, Germany; ∥ International Institute for Sustainability with Knotted Chiral Meta Matter (WPI-SKCM^2^), Hiroshima University, Hiroshima 739-8526, Japan; ⊥ Department of Chemical Engineering and Biotechnology, 2152 University of Cambridge, Cambridge CB3 0AS, U.K.; # Innovative Center in Digital Molecular Technologies, Yusuf Hamied Department of Chemistry, University of Cambridge, Cambridge CB2 1EW, U.K.

**Keywords:** cellulose nanocrystals, particle morphology, scanning nanobeam electron diffraction, small angle x-ray scattering, cholesteric liquid crystals

## Abstract

Cellulose nanocrystals (CNCs) are elongated nanoparticles derived from natural cellulose, with potential applications ranging from rheological modifiers and emulsion stabilizers to photonic pigments and sensors. For most applications, precise control over CNC morphology and surface chemistry is essential, but the relationship between process parameters, CNC characteristics, and their resulting behavior is poorly understood. Here, we investigate the impact of centrifugation and ionic strength on CNC morphology after dialysis using transmission electron microscopy, small-angle X-ray scattering, and scanning electron diffraction. We find that the centrifugation step commonly applied during CNC purification promotes the formation of compact composite nanoparticles made of aligned crystallites, referred to as “bundles,” that are associated preferentially along their hydrophobic faces. In stark contrast, transient exposure to high ionic strength leads to fractal-like, irregular composite nanoparticles. We then examine the consequence of these morphological differences on the cholesteric self-organization of the CNCs: aligned bundles reduce the cholesteric pitch in suspension, causing a blue-shift in the color of dish-cast photonic films, while misaligned particles promote gelation, producing colorless films. This study reveals the importance of sample history, in particular, the often-disregarded purification steps, on CNC characteristics and their ensemble behavior, thereby unlocking new routes for tailoring this promising nanomaterial.

## Graphical abstract

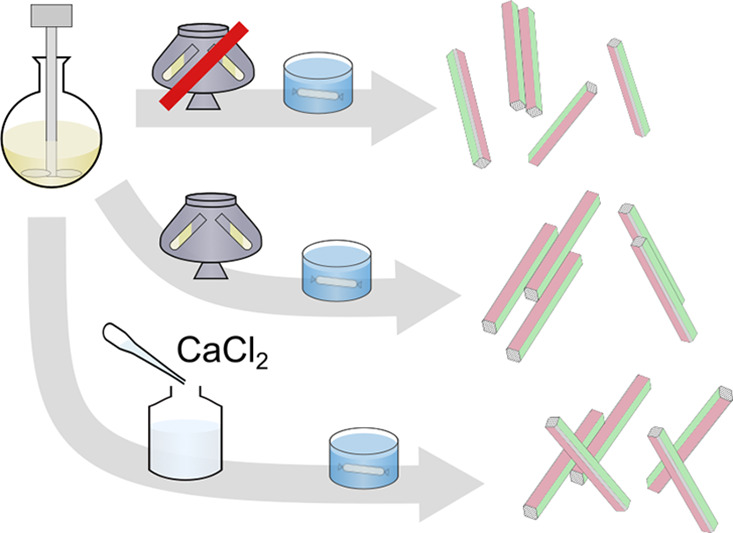

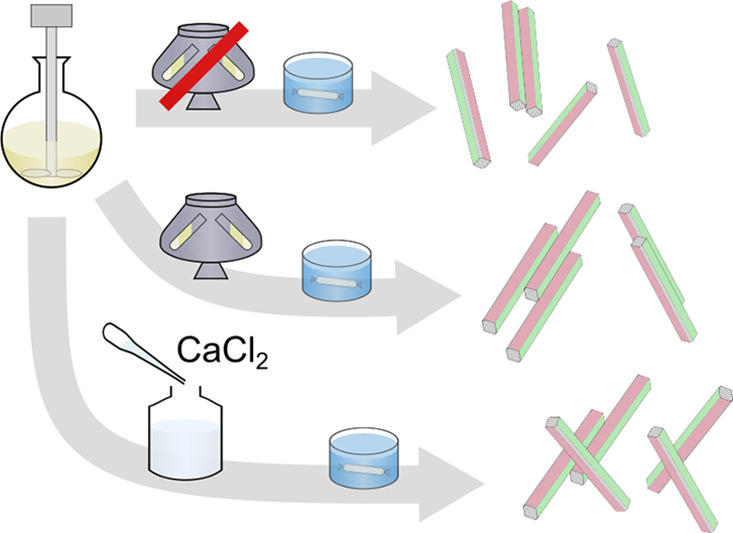

## Introduction

Cellulose nanocrystals (CNCs) are elongated crystalline nanoparticles derived from native cellulose.^1,2^ CNCs have recently attracted significant interest as a sustainably sourced nanomaterial for numerous potential applications.^3^ At present, the most industrially relevant method used for CNC extraction is the hydrolysis of wood cellulose with concentrated sulfuric acid (∼10 M). During this process, the cellulose fibers are degraded and disassembled into nanoscale fragments, while sulfate half-ester groups (−OSO_3_H) are grafted onto the exposed surfaces.^4,5^ Then, the reaction is quenched via dilution with water and the nanoparticles are purified. At the laboratory scale, this purification process usually consists of several rounds of centrifugation and redispersion in deionized water, which allows for the separation of the acidic supernatant from the CNC pellet, followed by dialysis against deionized water to remove excess ions. The resulting CNC suspension is a colloidally stable mixture of isolated cellulose crystallites and composite multicrystallite particles.^6–8^

Among the composite particles, the presence of raft-like particles made of aligned crystallites—often referred to as bundles—influences key characteristics of the final suspension. For example, an increasing proportion of CNC bundles has been correlated with a decrease in the cholesteric pitch and a narrowing of the concentration range of the biphasic regime.^8^ Moreover, the presence of bundles could also modify the overall behavior of CNCs as Pickering agents, due to their distinctive morphology and amphiphilicity.^9–11^ On this basis, it has been proposed that the crystallites that make up the bundles are preferentially associated along a specific crystal plane.^9–11^ Yet, the substructure of these objects has never been observed, leaving it unclear which specific crystal face, if any, might be involved in a potential preferential orientation.

Whether these laterally associated composite particles predate the hydrolysis (and are therefore related to the source),^6^ or are mostly formed through aggregation during the production process remains an open question. Conventional DLVO theory suggests that the high ionic strength conditions during the hydrolysis and the initial rounds of centrifugation (*i.e.*, [H_2_SO_4_] ≈4–10 M) should cause irreversible aggregation of the CNCs. Nevertheless, CNC pellets can be readily redispersed upon lowering the ionic strength and are assumed to do so spontaneously during dialysis, with final suspensions exhibiting very little sedimentation. This discrepancy with theory is rarely mentioned in the literature, with few studies investigating the impact of hydrolysis conditions (*e.g*. acid concentration, temperature) on the CNC morphology and properties,^2^ and fewer studies considering the influence of the suspension history after hydrolysis.^12–15^ This likely results in the oversight of experimental factors that are crucial in determining the CNC characteristics. For example, little attention has been given to the centrifugation parameters used during purification (*i.e.*, relative centrifugal force, duration), with reported conditions ranging from intense centrifugation (*e.g.* 20,000*g* for 20 min) to none at all,^16–18^ and with studies often not even specifying the parameters used.

In this work, we investigate the impact of the post-hydrolysis centrifugation step on the morphology of CNCs and compare it with the irreversible aggregation induced by divalent cations. Using dynamic light scattering (DLS), transmission electron microscopy (TEM), small-angle X-ray scattering (SAXS), and viscometry, we show that CNC purification by centrifugation favors the formation of bundles, *i.e.*, compact laterally aligned composite particles (Figure 1a). We further show by scanning nanobeam electron diffraction (SNBED) that these bundles are preferentially associated through their hydrophobic faces. Conversely, destabilization by exposure to calcium chloride (CaCl_2_) leads to an irreversible increase in the CNC size due to the formation of randomly associated fractal-like composite particles held by Ca^2+^ cross-linking (Figure 1b). We then investigated the consequences of aggregation on the cholesteric self-assembly behavior. We found that increasing the presence of bundles reduces the cholesteric pitch, resulting in a blue-shift of the films obtained by dish-casting the suspension, while the presence of irregular particles made using CaCl_2_ instead promote gelation at a lower concentration, leading to colorless films (Figure 1c).

**Figure 1:**
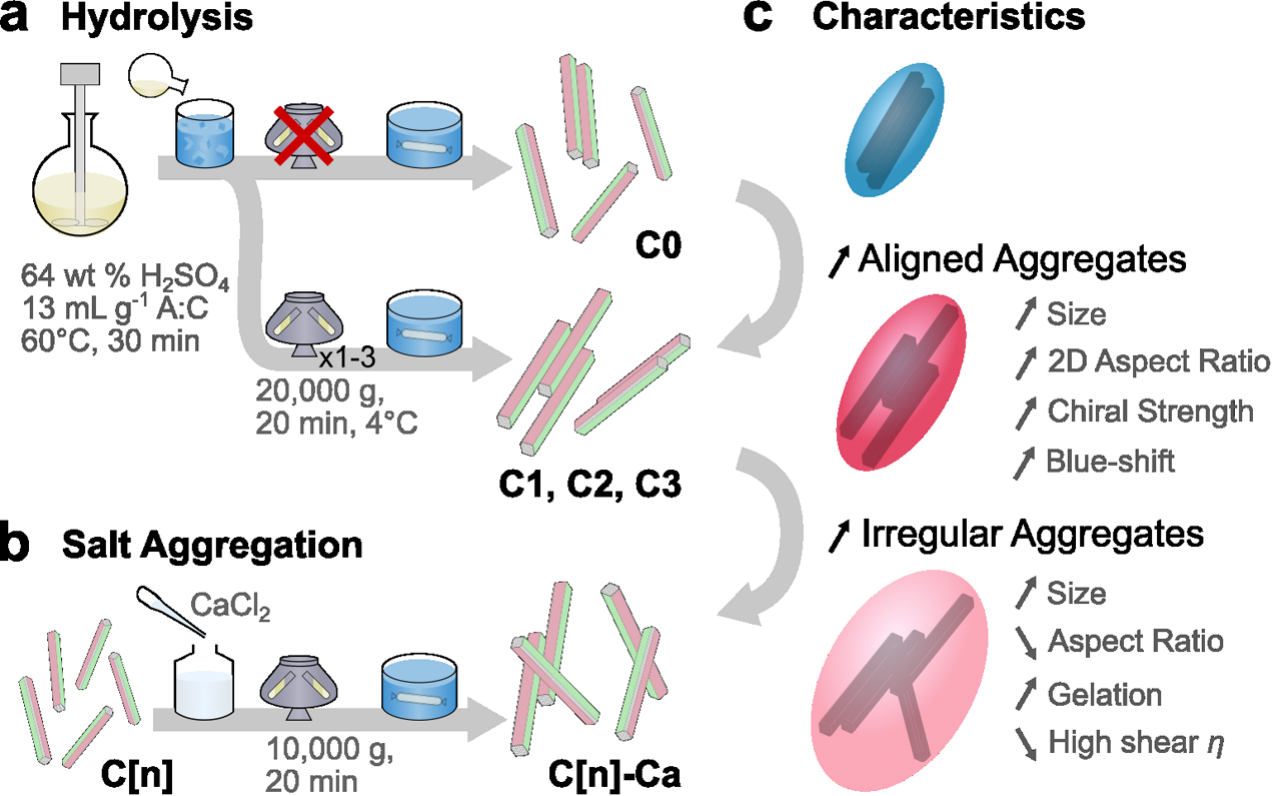
Scheme summarizing the preparation of (a) never centrifuged (**C0**) and centrifuged (**C1–C3**) CNCs, and (b) salt aggregated CNCs subsequently produced by CaCl_2_ addition followed by centrifugation (**C0–Ca** and **C3–Ca**), and (c) corresponding key differences in CNC characteristics relative to **C0**.

## Results and Discussion

### Self-Limiting CNC Aggregation by Centrifugation

To explore the influence of post-hydrolysis centrifugation on CNC morphology, cotton-derived cellulose was hydrolyzed into CNCs according to the conditions summarized in Figure 1a and detailed in the Methods section. After quenching the hydrolysis reaction, part of the mixture was isolated and never centrifuged (**C0**), while the remaining mixture was subjected to multiple cycles of centrifugation and redispersal of the pellet in ultrapure water. Aliquots of the mixture were isolated after one, two, or three cycles of centrifugation and redispersion, resulting in samples **C1**, **C2**, and **C3**, respectively.

The effect of centrifugation on the CNC size after dialysis was investigated by measuring the Z-average hydrodynamic diameter (*D*_H_) of each suspension by DLS. As shown in Figure 2, the first round of centrifugation (from **C0** to **C1**) led to a significant increase in *D*_*H*_, while subsequent rounds (from **C1** to **C2** or from **C2** to **C3**) had no significant effect. This indicates that the first centrifugation step applied during CNC purification leads to composite particle formation.

**Figure 2:**
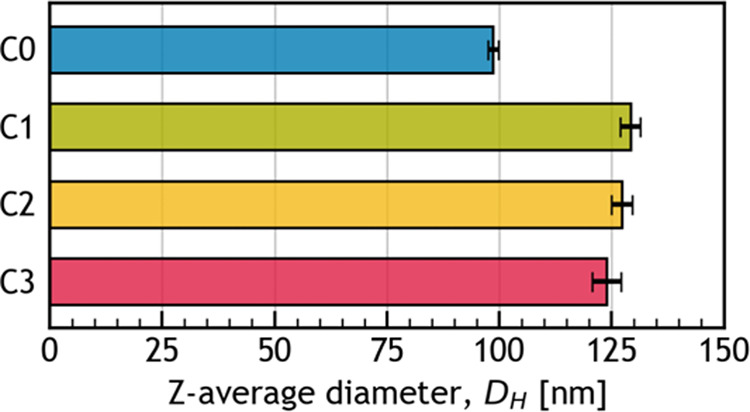
Z-average diameter (*D*_*H*_) of the never centrifuged CNCs (**C0**) and CNCs centrifuged once (**C1**), twice (**C2**), or three times (**C3**), as determined by dynamic light scattering (DLS).

To evaluate the colloidal stability of the CNCs, the surface charge for each suspension was determined by conductometric titration against sodium hydroxide. The centrifuged (**C1**, **C2**, **C3**) and never-centrifuged CNCs (**C0**) all exhibited a similar amount of sulfate half-ester groups on their surface (264 ± 5 mmol kg^–1^). The similar surface charge exhibited by all the samples suggests that the formation of composite particles did not cause the –OSO_3_H groups to become buried between internal crystallite interfaces. These typical surface charge values, along with the high ζ-potential absolute value (ζ ≈ −49 mV, see Table S1), are indicative of good colloidal stability at low ionic strength.

However, the ionic strength (*I*) of the medium during the first and second centrifugation steps (with [H_2_SO_4_] > 0.1 M) is significantly greater than the thresholds sufficient to induce aggregation of sulfated CNCs (typically reported to be <0.03 M).^19–21^ Consequently, the CNCs are expected to be colloidally unstable during both these steps. Yet, no further size increase between **C1** and **C2** was observed after dialysis. This suggests that centrigugation under high ionic strength triggers irreversible CNC aggregation, but only up to a certain size limit, likely reached during the first centrifugation. Further aggregation beyond this point appears reversible, as it can be undone by lowering the ionic strength through extensive dialysis against water. Moreover, all the suspensions previously experienced a stronger ionic strength environment during the hydrolysis, indicating that the irreversible formation of composite particles during centrifugation cannot be attributed solely to electrostatic destabilization.

### Irreversible CNC Aggregation by Salt Addition

The role of electrostatic destabilization in the irreversible formation of composite CNC particles was investigated by increasing the ionic strength of CNC suspensions, followed by redispersion at low ionic strength to retain only irreversible aggregates. For these experiments, the **C0** and **C3** suspensions were pH-neutralized with sodium hydroxide and concentrated to yield **C0–Na** and **C3–Na**, respectively.

The protocol for transiently increasing the ionic strength consisted of four steps, as summarized in Figure 3a: (i) preparation of a CNC suspension at fixed ionic strength (*I*_*agg*_) and CNC weight fraction (*w*_*agg*_ = 6.5 wt %), (ii) centrifugation (10,000*g* for 20 min), (iii) redispersion by mixing, and (iv) dilution to 0.1 wt % CNC and with an ionic strength as close as possible to 1 mM for DLS measurement. These parameters were selected following a series of experiments to optimize the protocol (see Section S2.1, Table S2, Figures S2 and S3). Interestingly, the size of the irreversible aggregates increased during the first hour of exposure to elevated ionic strength, after which the size plateaued (see Table S2 and Figure S3).

**Figure 3:**
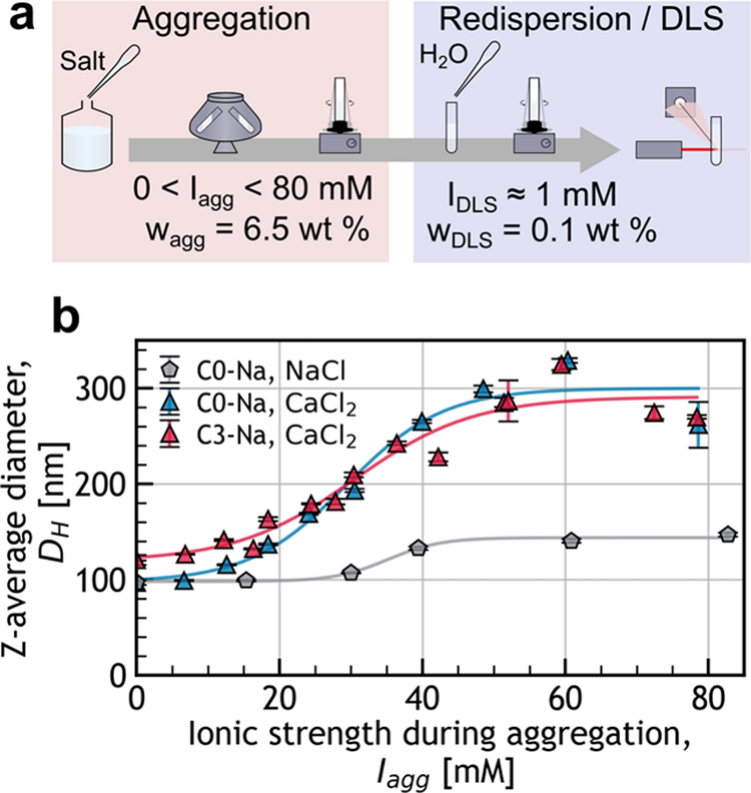
(a) Scheme summarizing the calcium aggregation process and (b) the impact of salt type (NaCl vs CaCl_2_) and transiently raised ionic strength (*I*_*agg*_) at fixed weight fraction (*w*_*agg*_ = 6.5 wt %) during the aggregation step on the resulting Z-average diameter (*D*_*H*_), as measured after dilution to 0.1 wt % in an ionic strength as close as possible to 1 mM. The lines of best fit are presented as visual guides and were obtained from a logistic function.

In an initial experiment, the never-centrifuged CNCs were mixed with NaCl at varying transient ionic strengths (0 ≤ *I*_*agg*_ ≤ 80 mM). As shown in Figure 3b, no change in *D*_*H*_ was observed after the centrifugation of **C0–Na** without added salt (*I*_*agg*_ = 0). This indicates that the size increase observed between **C0** and **C1** was only made possible by the high ionic strength of the medium. Then, a moderate increase of particle size was first observed around *I*_*agg*_ = 30 mM before quickly reaching a plateau of ∼145 nm for *I*_*agg*_ ≥ 50 mM (see also Figure S4 for corresponding intensity-based DLS size histograms). This suggests that the size of the irreversible aggregates formed by exposure to high ionic strength is self-limited, in agreement with the previous DLS measurements (Figure 2). Our findings contrast with a previous study reporting that NaCl-induced aggregation of CNCs was reversible upon dialysis against deionized water.^20^ However, since the CNCs in that study were already centrifuged during purification, we suspect that they had already reached their maximum self-limiting size prior to salt-induced aggregation.

To determine whether this size limit could be overcome, we investigated the effect of a divalent cation by using calcium chloride (CaCl_2_) instead of NaCl. In this case, the CNCs exhibited a much greater increase in *D*_*H*_ than that observed with NaCl at equivalent *I*_*agg*_ (Figure 3b). The *D*_*H*_ values followed a sigmoid-like curve with *I*_*agg*_, starting to increase from approximately 100–120 nm at *I*_*agg*_ = 12 mM before reaching a plateau around 290 nm above *I*_*agg*_ = 50 mM. Above *I*_*agg*_ = 40 mM, the variability of the measurements seemingly increased significantly, and macroscopic gel-like objects were visible in the sample cuvette for *I*_*agg*_ ≥ 50 mM. The destabilization with CaCl_2_ was repeated using centrifuged CNCs (**C3–Na**), leading to similar results (Figure 3b). Although the particle sizes were initially slightly larger than the never-centrifuged sample, both samples exhibited comparable sizes for *I*_*agg*_ ≥ 20 mM.

These results indicate that the size of the irreversible aggregates is dependent on the cation used, in agreement with previous studies comparing CNC aggregation induced by monovalent or polyvalent cations.^4,20–23^ In the present case, the difference could be attributed to the irreversible formation of calcium cross-links between CNCs. Furthermore, the similar trends observed for **C0–Na** versus **C3–Na** suggest that the mechanism of calcium-induced aggregation is different from centrifugation-induced aggregation. The two methods thus provide independent pathways to produce composite CNCs.

### Dialyzed Calcium-Aggregated CNC Suspensions

To explore the differences in particle characteristics between the two aggregation pathways, **C0–Na** and **C3–Na** were centrifuged in the presence of calcium chloride (*I*_*agg*_ = 40 mM) followed by dialysis against water, to respectively produce **C0–Ca** and **C3–Ca**, collectively referred to as Ca-CNCs (see protocol illustrated in Figure 1b). Elemental analysis of **C3–Ca** yielded a calcium content of 133 mmol kg^–1^, corresponding to exactly half of the sulfate half-ester groups measured for its parent sample (**C3**) (264 ± 5 mmol kg^–1^, see Table S1). Given the divalent character of Ca^2+^ ions, this corresponds to 266 mequiv kg^–1^ of Ca^2+^, indicating that all the charged groups on the surface of **C3–Ca** were in the form of calcium salts and that the charged groups were not hidden by the calcium-induced destabilization.

The hydrodynamic diameter of these Ca-CNCs was confirmed to be substantially greater than for **C0** and **C3** (Table S1), and a comparison of the DLS-derived count rate versus Z-average diameter suggests that the Ca-CNCs are considerably less compact than the **C0, C1, C2**, and **C3** samples (Figure S1 and Section S1.1). However, as a single, ensemble-averaged quantity, the hydrodynamic size provided by DLS measurements cannot distinguish more subtle morphological differences between samples.

### CNC Particle Morphology (TEM)

TEM was used to compare the morphological properties of the individual CNC particles, as exemplified in Figure 4a. Visual inspection of the images revealed a variety of particle types in each sample, including composite particles constituted of aligned subunits (bundles) and irregular composite particles, which are both commonly reported for wood and cotton-derived CNCs.^6,8^ Notably, **C0–Ca** and **C3–Ca** appeared to contain a higher proportion of larger irregular composite particles compared to **C0** and **C3**.

**Figure 4:**
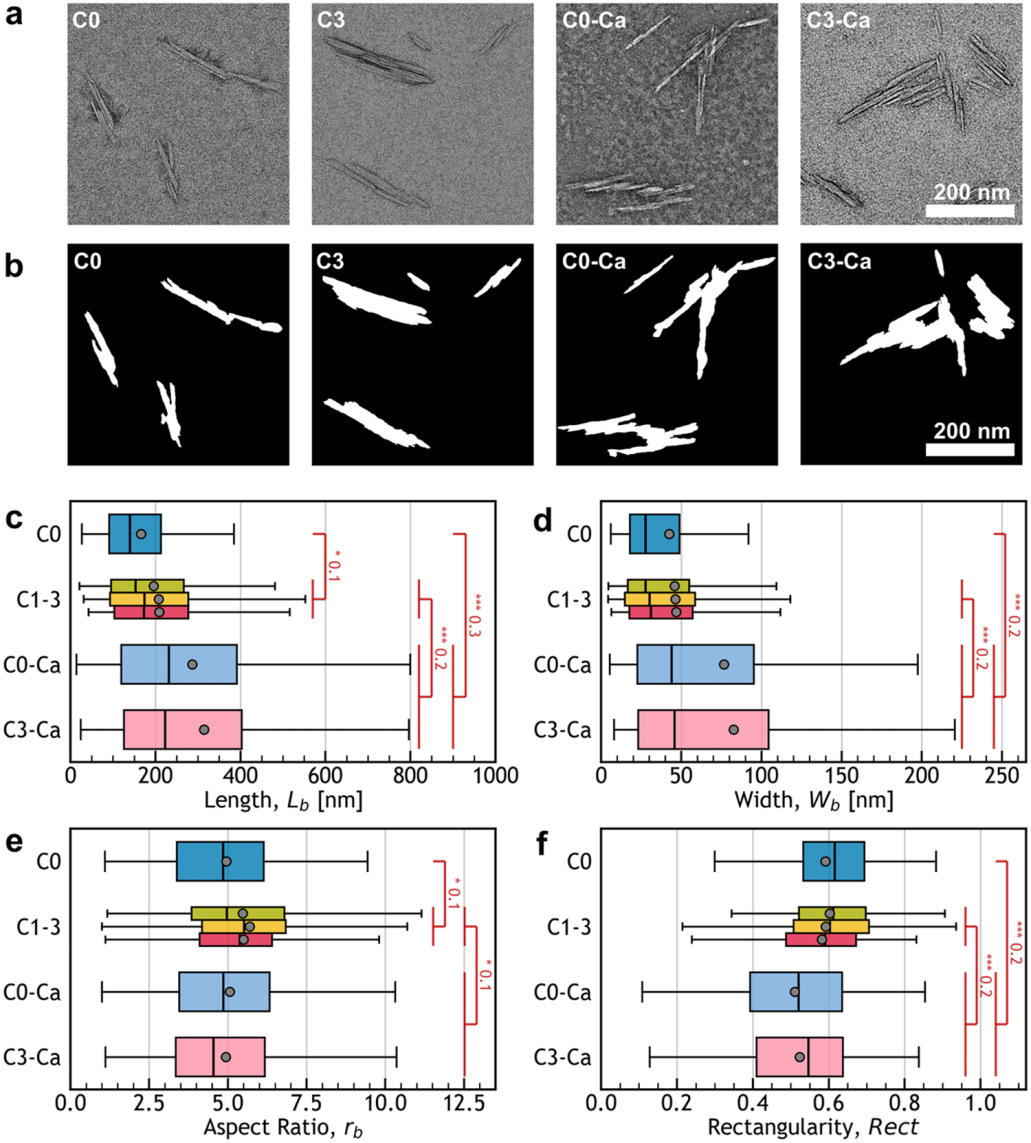
Analysis of the CNC morphology from TEM images. (a) Typical objects observed by TEM and (b) examples of the corresponding contoured shapes used for dimension extraction. (c–f) Boxplots of the morphological parameters extracted: (c) bounding box lengths (*L*_b_), (d) bounding box widths (*W*_b_), (e) bounding box aspect ratios (*r*_b_), and (f) rectangularity (Rect). Significance of the pairwise Mann–Whitney *U* test is indicated by the following *p*-value thresholds: *, 0.05 > *p* > 0.01; **, 0.01 > *p* > 0.001; and ***, *p* < 0.001. The importance of the effect is indicated by the absolute point biserial coefficient (number in red), with values of 0.5, 0.3, and 0.1 often considered as obvious (large), subtle (medium), and merely statistical (small) effects, respectively (see Section S4.2 for more details). Gray-filled circles indicate the average values and outliers are not displayed; full data are presented in Figures S5 and S6.

To quantitatively compare the particles, their morphological properties were extracted. In the literature, the presence of composite particles leads to inconsistencies regarding what is considered an individual CNC during particle characterization, leading to significant human operator bias during image analysis.^2^ Considering each discrete object on the TEM grid as a single CNC, regardless of whether it appears as a single crystallite or as a composite object formed by multiple overlapping crystallites, minimizes the operator bias from particle selection and produces morphological distributions consistent with ensemble-averaged size values.^8^ Using this approach, the outline of more than 225 particles per sample were traced, as exemplified in Figure 4b.

The distributions for the bounding box length (*L*_*b*_) and width (*W*_*b*_) of the CNC outlines are shown as box plots in Figure 4c,d. As typically observed for CNCs, both parameters exhibited high variance, making it challenging to determine whether the observed differences in mean values are statistically significant. Moreover, while the histograms for *L*_*b*_ and *W*_*b*_ are often modeled as log-normal distributions,^6,24^ a thorough investigation revealed that most samples in this study displayed signs of deviation from log-normality (see Figure S5, Table S3, and Section S3.2). Therefore, the series were compared pairwise using the nonparametric Mann–Whitney *U* test (see Figure 4c,d and Section S4.2), which determines the statistical significance of a difference between the means of two distributions of arbitrary type.^25^ The magnitude of the difference in mean values was also quantified using the point biserial correlation coefficient *r* (detailed calculation in Section S4.2).^26^

As indicated in Figure 4c,d, the never-centrifuged sample (**C0**) exhibited significantly shorter particles than the centrifuged CNCs (**C1**–**C3**) while the centrifuged CNCs displayed similar lengths and widths. Similarly, the Ca-CNCs (**C0–Ca** and **C3–Ca**) exhibited similar dimensions that were significantly longer and wider than all the other CNCs. These trends are in agreement with the previous DLS measurements.

The morphology of the particles was further examined by calculating the bounding box aspect ratio (*r*_*b*_) and rectangularity (*Rect*) defined respectively as

1 $$r_{b}=\frac{L_{b}}{W_{b}}$$

and

2 $$\mathrm{Rect}=\frac{A}{L_{b}W_{b}}$$

where *A* is the outline area.^8^ The distributions for *r*_*b*_ and *Rect* for each sample were investigated graphically and statistically. As presented in Figure 4e,f, *r*_*b*_ and *Rect* followed skewed normal distributions for all samples (see Figure S6 for histograms and Table S3). Compared to **C1**, **C2**, and **C3**, **C0** exhibited a lower aspect ratio but a similar rectangularity, while Ca-CNCs display both a lower aspect ratio and a lower rectangularity.

Cryo-TEM of the CNC suspensions was used to estimate whether the observed morphologies originate from drying artifacts arising from standard TEM grid preparation.^27^ In the cryo-TEM images, **C0** and **C3** mainly appeared laterally associated with rare occurrences of irregular composite particles, whereas **C3–Ca** exhibited significantly more irregular objects (Figure S9). As such, these observations qualitatively confirm the divergence of morphology between the different samples determined from the standard TEM analysis.

In summary, the morphological parameters extracted from TEM images for **C0**, **C1**, **C2**, and **C3** indicate that centrifugation during purification leads to the irreversible formation of composite particles while retaining a similar shape. For elongated flat particles, this scaling infers the formation of compact composite particles constituted of aligned subunits (*i.e.*, bundles). Moreover, the comparable morphological parameters of centrifuged CNCs (**C1**, **C2**, **C3**) further suggest that the formation of bundles occurred only during the first round of centrifugation (**C0** to **C1**) and was likely due to the presence of a saturation point beyond which particle association is negligible.

Concerning the CNCs aggregated with CaCl_2_ (**C0–Ca** and **C3–Ca**), we hypothesize that they underwent random and irreversible association of crystallites into fractal-like irregular composite particles cross-linked by calcium ions. For charged elongated rods, extended DLVO theory predicts that crossed association offers a lower energetic barrier to overcome compared to parallel association.^28,29^ Therefore, despite the parallel association being more stable, crossed association is favored upon rapid aggregation, leading to bigger and less elongated particles. Moreover, this crossed-association mechanism is favored at high ionic strength,^30^ and was previously observed for charged CNCs.^21,31,32^

### CNC Ensemble Morphology (SAXS)

To validate the trends in individual particle morphology observed from TEM images, we characterized the same samples in suspension using Small Angle X-ray Scattering (SAXS). The SAXS spectra were collected over 0.008 ≤ *q* ≤ 0.247 nm^–1^, corresponding to distances in real space between 750 and 30 nm (∼2π/*q*). The corresponding concentration normalized intensities, *I*_*c*_ *(q)*, are presented in Figure 5a. At very low *q* (*q* < 0.013 nm^-1^), **C0**, **C3**, **C0–Ca** and **C3–Ca** all exhibited a lack of linearity of ln [*I*(*q*)] as a function of q^2^. This was previously attributed to CNC size polydispersity,^6^ and prevents reliable extraction of radii of gyration (*R*_g_) from a Guinier analysis. In the Porod regime (0.06 ≤ *q* ≤ 0.2 nm^–1^), the samples exhibited power law exponents of −1.9, −2.0, −2.1, and −2.1 for **C0**, **C3**, **C0–Ca** and **C3–Ca** respectively. This suggests a rather elongated but flattened shape with increasing degree of branching in this sequence. A similar trend was previously observed in the same *q* region upon increasing CNC aggregation through salt addition.^31^

**Figure 5:**
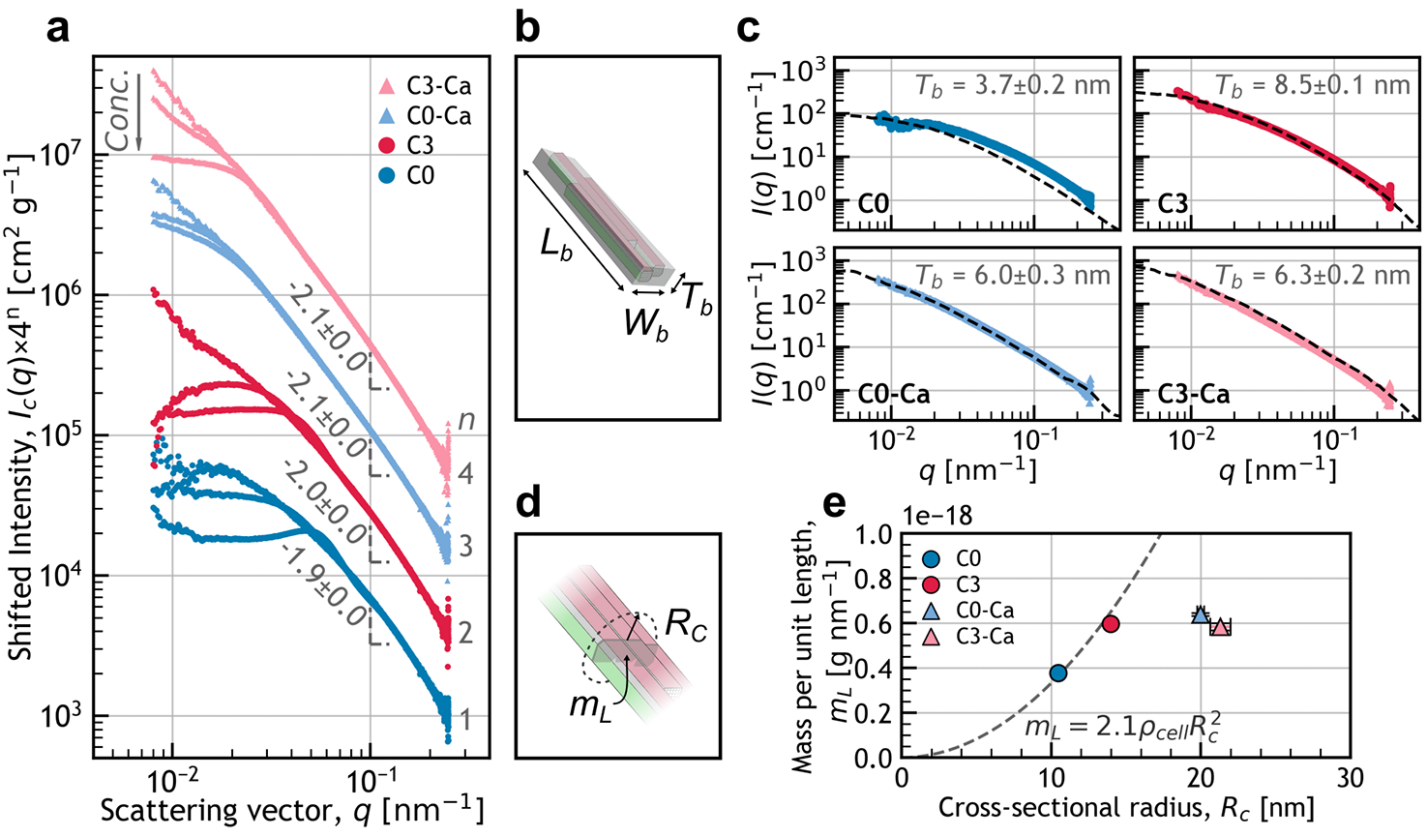
SAXS analysis of the CNCs and extracted morphological parameters. (a) Concentration normalized intensity, *I*_c_(*q*) at different concentrations (*Conc.*, starting from the bottom **C0**: 1.0, 0.5, and 0.1 wt %; **C3**: 1.0, 0.5, and 0.1 wt %; **C0–Ca**: 0.77, 0.5, and 0.1 wt %; and **C3–Ca**: 1.0, 0.5, and 0.1 wt %, all rescaled vertically for clarity), and the corresponding Porod exponents (measured at 0.06 ≤ *q* ≤ 0.2 nm^–1^). (b) Rectangular prism model used to compute SAXS intensity profiles from the TEM-extracted length (*L*_b_) and width (*W*_b_), and the corresponding best fitting thickness (*T*_b_). (c) Scattering intensity profiles *I*(*q*) at 0.1 wt % for each sample, reported with best thickness and corresponding best fitting model (black dashed line). (d) Schematic illustrating the particle cross-sectional mass (*m*_*L*_) and cross-sectional radius of gyration (*R*_*c*_) extracted from the cross-sectional Guinier analysis. (e) Corresponding evolution of *m*_L_ as a function of *R*_c_, and best fit of *m*_*L*_ *= a* *ρ_cell_* *R*_*c*_^2^ for **C0** and **C3**. See Section S4 for more details.

At low concentration, the SAXS intensity profile of a sample is mainly determined by the shape of the particles in suspension. To extract this morphological information, a SAXS intensity profile was modeled for each sample by generating populations of polydisperse rectangular prismes, as illustrated in Figure 5b. The lengths and widths of the objects were randomly generated from the sample size distributions obtained by TEM, while their thickness (*T*) was set to a single, arbitrary value (see Section S4.1).^6^ For each sample, this process was repeated over a range of T values to identify the thickness *T*_*b*_ that best matched the considered experimental SAXS curve (see Figure S10 and Section S4.2). The resulting *T_b_* for **C0**, **C3**, **C0–Ca** and **C3–Ca** were 3.7, 8.5, 6.0 and 6.3 nm respectively. The corresponding modeled intensity profiles are shown in Figure 5c. The excellent agreement between the modeled and experimental SAXS profiles indicates that the dimensions extracted from TEM are representative of the particles in suspension.

The extracted *T*_*b*_ value for C0 (*T_b_* = 3.7 nm) appears unexpectedly small compared to previous studies on cotton CNCs and crystallites.^6,31^ This likely reflects a biais introduced by the modeling approach. Indeed, applying the bounding box dimensions to a rectangular prisms model for samples containing a significant proportion of irregular composite particles (*i.e.* a low rectangularity) leads to an overestimation of the particle volume (see Figure 4b, f). Since the SAXS intensity scales with the actual volume (or mass) of cellulose contained in each particle (at a given *Φ*), a lower apparent thickness *T*_*b*_ compensates for such volume overestimation.

Interestingly, although **C3** exhibited larger bounding box dimensions than **C0**, is also had a greater thickness (*T_b_* = 8.5 vs. 3.7 nm). This suggests that **C3** contains larger particles with a comparable rectangularity, consistent with a higher proportion of bundles in this sample. In contrast, **C0–Ca** and **C3–Ca** exhibited intermediate thickness values (*T_b_* = 6.0 and 6.3 nm respectively), despite displaying even larger bounding box dimensions. This discrepancy likely results from a greater proportion of irregular composite particles in these samples, as indicated by their lower rectangularity (see Figure 4f)

To compare the cross-sectional density of the CNCs while minimizing assumptions, a cross-sectional Guinier analysis at intermediate *q* was conducted by assuming a cylindrical particle shape (see Figure S11 and Section S4.3). The corresponding cross-sectional radius of gyration (*R*_*c*_) and the cross-sectional particle mass (*m*_*L*_, g nm^–1^) are shown in Figure 5d,e. The *R*_*c*_ increased from **C0** to **C3** and then further to Ca-CNCs, indicating an increase in the cross-section of the CNCs in this order. For a compact particle, an increase in cross-section is expected to cause a proportional increase of *m*_*L*_ with *R*_*c*_^2^. Fitting of *m*_*L*_ against *ρ*_*cell*_ *R*_*c*_^2^ for **C0** and **C3** leads to a proportionality factor of 2.1 ± 0.1, supporting that these samples have a similar compactness, in agreement with bundle formation upon centrifugation.

Concerning the Ca-CNCs samples, they exhibited *m*_*L*_ values that are similar between each other and comparable to the one of **C3**. However, their *R*_*c*_ was much larger, revealing a distinctive decrease in cross-sectional density upon calcium-induced aggregation, in accordance with the formation of randomly associated particles. Interestingly, **C0–Ca** and **C3–Ca** presented a similar cross-sectional density, despite their respective parent samples having different morphologies. Yet, if calcium-induced aggregation only caused random association of the particles, then **C0–Ca** should contain fewer bundles than **C3–Ca** and therefore exhibits a lower cross-sectional mass and apparent *T_b_*, which is not observed here. Instead, it seems that calcium-induced aggregation also triggers some lateral association between crystallites, within the irregular particles, to the same extent as the centrifugation during purification, rendering **C0–Ca** and **C3–Ca** structurally comparable.

Overall, the morphological parameters extracted from the SAXS analysis are consistent with the TEM and DLS analysis. This further supports the formation of compact laterally associated particles upon centrifugation in sulfuric acid, and the formation of randomly associated fractal-like particles upon calcium-induced aggregation. Moreover, this ensemble measurement demonstrates that the reported morphological differences between these samples are statistically significant in suspension, despite the CNC polydispersity.

### Relative Orientation of Crystallites within CNCs (SNBED)

The morphological characterization techniques discussed above only consider the overall particle morphology and the alignment of the long axes of the crystallites within the composite particles. However, the relative orientation of the crystallite cross-section with respect to adjoining crystallites can also have an important impact on the CNC properties. In cotton fibers, native crystallites are mainly found in the cellulose Iβ crystalline allomorph and are believed to possess approximately hexagonal cross sections, as illustrated in Figure 6a.^33^ Consequently, the vast majority of the exposed crystal surfaces corresponds to the (110) and (1–10) planes, with the remaining surfaces corresponding to the (100) plane. The greater density of hydroxy groups on the (110) and (1–10) planes is expected to make these faces more hydrophilic than the (100) plane, which is therefore described as “hydrophobic” in comparison.

**Figure 6:**
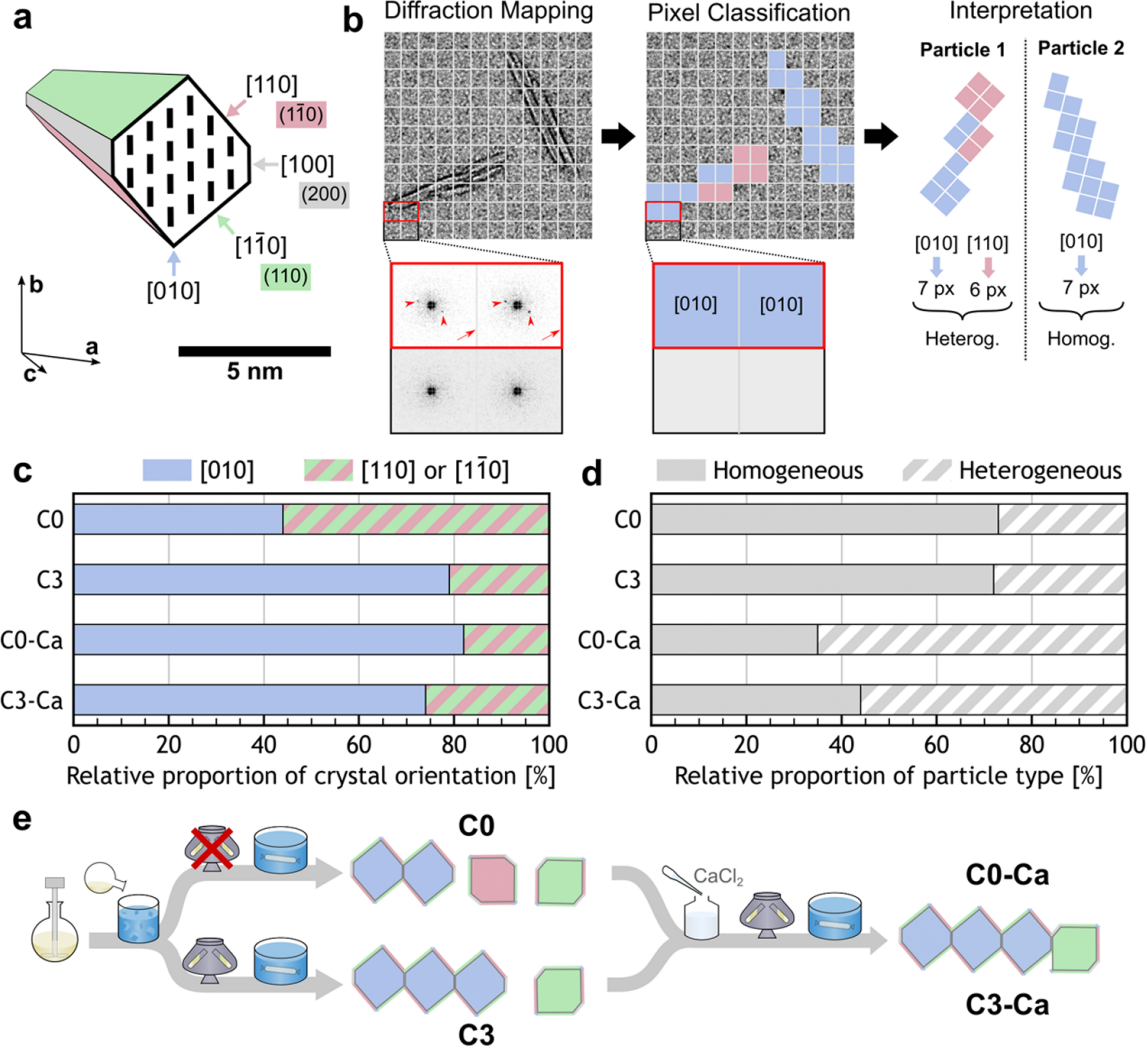
Local orientation of the crystallites constituting the cellulose nanocrystals probed by scanning nanobeam electron diffraction (SNBED). (a) Cross-section of a native cellulose Iβ monocrystal from cotton, with the crystal zone-axes directions and their corresponding crystal planes labeled. (b) Illustration of the diffraction pattern mapping process, with pixel classification (illustrative stained TEM image) according to crystal orientation ([010] in blue, [110] in pink, or [1–10] in green), and particle classification with particles displaying a mix of crystal orientations classified as heterogeneous (**Particle 1**) and pixels from particles displaying only one orientation classified as homogeneous (**Particle 2**). The pixels have a dimension of 25 × 25 nm^2^. (c) Relative proportion of cellulose crystal plane orientations (based on pixel number) and (d) relative proportion of particle type for the different CNC samples. (e) Schematic summarizing the preparation of the different CNC samples and the resulting impact on the crystallite orientations and particle types observed by SNBED.

The relative orientation of the cross-section of the crystallites within composite CNCs cannot be determined by conventional morphological characterization such as TEM or AFM. Therefore, we characterized our samples using scanning nanobeam electron diffraction (SNBED), a method of four-dimensional scanning transmission electron microscopy (4D-STEM). This technique utilizes a focused electron beam to obtain the local 2D electron diffraction (ED) patterns for each position scanned across a TEM grid, as illustrated in Figure 6b.^34^ In this work, each ED pattern was collected by an electron beam probe with a diameter of 25 nm, with all crystallites within the probe diameter contributing to the obtained diffraction information. Each scan position containing an ED pattern (constituting a “pixel”) was indexed according to the cellulose Iβ unit cell.^35^ This information was used to determine the local crystallographic orientation with respect to the incident electron beam, as illustrated in Figure 6b. Example SNBED data are provided in Figure S12.

The CNC diffraction patterns for **C0**, **C3**, **C0–Ca**, and **C3–Ca** could be divided into three distinct categories, corresponding to either the [010], [110], or [1–10] zone axes pointing normal to the TEM grid surface. None of the diffraction patterns corresponded to the [100] zone axis, indicating that crystallites were never observed with their hydrophobic faces oriented against the grid. This is likely due to either poor adhesion between the hydrophobic (100) plane and the hydrophilic glow-discharged carbon film used as the TEM grid, or cross-sectional anisotropy of the particles. The lack of observation of other crystallographic planes suggests the CNCs are relatively well-faceted in the planes corresponding to the observed zone axes.

Figure 6c shows the relative amount of each crystallite orientation for each sample as a proportion of the SNBED pixels for which diffraction patterns were observed. For the never-centrifuged CNCs (**C0**), the proportion of pixels corresponding to the [010] zone axis (pointing normal to the TEM grid) was *P*_[010]_ = 44%, with the remaining 56% divided between the [110] and [1–10] zone axes. In contrast, for the centrifuged CNCs (**C3**) and the Ca-CNCs (**C0–Ca** and **C3–Ca**), a strong majority of the measured pixels corresponded to the [010] zone axis (respectively *P*_[010]_ = 79, 82, and 74%). This observation was corroborated by selected area electron diffraction (SAED) conducted on the same suspensions on more densely deposited grids and on much larger areas (∼0.8 μm^2^) containing a large ensemble of particles, and averaged over multiple locations across the TEM grid (see Figure S13). These results indicate that for all the samples, the crystallites are more likely to be facing the TEM grid on the (010) crystal plane than on any other plane.

The greater *P*_[010]_ for **C3** compared to **C0** suggests that the formation of bundles increases the number of crystallites with their (010) plane facing the grid (Figure 6c). This observation cannot be explained simply by different relative interactions between specific crystal faces and the TEM grid (see discussion in Section S5.2). Instead, we propose that the greater *P*_[010]_ for **C3** relative to **C0** arises from the preferential association of the crystallites along their hydrophobic (100) plane when they join to form bundles, as illustrated in Figure 6e. Such a raft-like structure is more likely to settle on the grid along the (010) plane and would therefore exhibit a single homogeneous [010] zone axis diffraction pattern, despite being composed of multiple distinct crystallites.

Previous studies have postulated that crystallites in bundle-like CNCs are preferentially associated along these hydrophobic faces.^7,10^ Such preferential orientation is plausible, as it would lead to a minimization of the free energy through a lowering of the proportion of hydrophobic faces exposed with water. Indeed, simulations showed that the work of adhesion between two (100) surfaces in water is greater than between two (110) surfaces or between a (100) and a (110) surface.^36^ Moreover, this association mechanism is consistent with the preservation of the number of charged groups per mass upon bundle formation from **C0** to **C3** (see Table S1), as an association along the hydrophobic faces (expected to be noncharge-bearing) would maintain the number of exposed charged groups. The impact of this preferential association on the tensioactive ability of the CNCs was investigated through pendant drop experiments of aqueous CNC suspensions in oil (see Figure S15 and Section S6). Compared to **C0–Na**, **C3–Na** displayed a statistically significant but small decrease in interfacial tension (∼2 mN m^–1^). This difference likely arises from the balance of competing effects including amphiphilicity and cross-sectional morphology of the particles, but also surface coverage and interparticle interactions.

The SNBED data set was further analyzed to assess the heterogeneity of the crystalline orientations within individual CNCs. For this, the data were classified based on the number of extracted crystal orientations within an individual particle: particles composed of pixels displaying two or more distinctive crystallographic orientations were classified as “heterogeneous” (illustrated by particle 1 in Figure 6b), while particles containing pixels all sharing the same crystallographic orientation were classified as “homogeneous” (illustrated by particle 2 in Figure 6b).

The relative occurrence of each particle type is shown in Figure 6d. For the never-centrifuged (**C0**) and the centrifuged (**C3**) samples, nearly three-quarters of the particles were homogeneous (73 and 72%, respectively). This further suggests that bundle-like CNCs are made of preferentially oriented crystallites. In contrast, much fewer homogeneous particles were observed for **C0–Ca** and **C3–Ca** (35 and 44%, respectively), which is consistent with these samples containing a large proportion of randomly associated crystallites.

The SNBED data for **C0–Ca** and **C3–Ca** were then compared to their parent samples **C0** and **C3**. Compared to **C3**, the**C3–Ca** exhibited a similar *P*_[010]_ with a higher proportion of heterogeneous crystal orientations (Figure 6c,d). These observations could be explained by the random association of new crystallites to preexisting raft-like particles, leading to an increase of heterogeneous particles with a similar *P*_[010]_ (Figure 6e). However, the *P*_[010]_ value for **C0–Ca** was substantially higher than for **C0**, but comparable to **C3** and **C3–Ca** (Figure 6c,d). This suggests that calcium-induced aggregation of never-centrifuged CNCs has the combined effect of reducing the proportion of hydrophobic planes (to the same extent as centrifugation-induced bundling in **C3**) while also forming heterogeneous particles (Figure 6e). This conclusion agrees with the similar cross-sectional density of **C0–Ca** and **C3–Ca** in the analysis of the SAXS data. This lateral association is also suggested by the size increase exhibited by CNC suspensions concentrated in the presence of NaCl (see Figure S17 and discussion in Section S8) and has been hypothesized to explain the formation of a stiffer network upon gelation of CNCs in the presence of additional cations.^23^

### Liquid Crystalline Ordering of the CNC Suspensions and Films

To determine whether the bundles formed by centrifugation display any enhanced chiral strength,^8^ the liquid crystalline properties of the different CNC suspensions were characterized. In suspension, CNCs spontaneously phase-separate into a denser anisotropic liquid crystalline phase and a lighter isotropic phase. With increasing CNC concentration, the proportion of anisotropic phase increases until the entire suspension becomes anisotropic. The volume fraction of the anisotropic phase (φ_*ani*_) as a function of the CNC volume fraction (Φ) for **C0–Na**, **C3–Na**, and **C3–Ca** is presented in Figure 7a. Compared to the never-centrifuged CNCs (**C0–Na**), the biphasic regime of **C3–Na** was narrower and shifted to lower concentrations. According to the Onsager model for achiral hard rods, this is indicative of rods having a larger aspect ratio.^37^ This is in accordance with our morphological analyses indicating that centrifuged CNCs exhibit an aspect ratio greater than or equal to the never-centrifuged CNCs.

**Figure 7:**
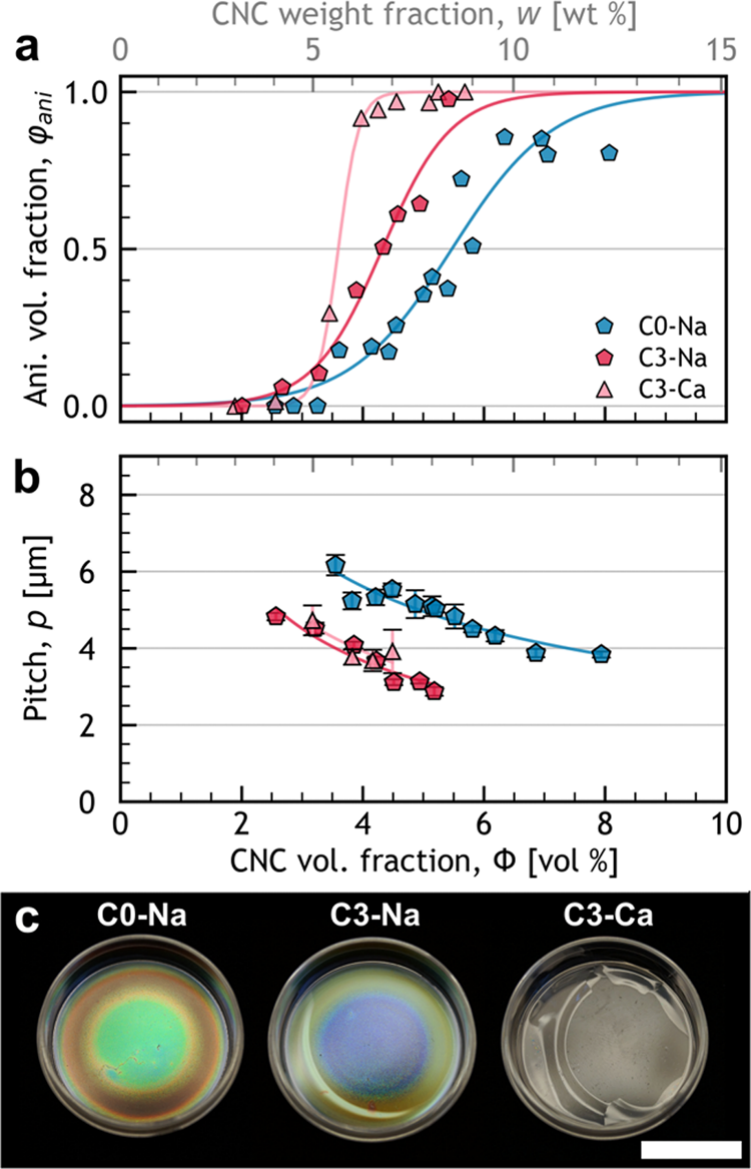
Liquid crystalline properties of different CNC suspensions: (a) volume fraction of the anisotropic phase (φ_ani_) and (b) pitch (*p*) as a function of total CNC volume fraction (Φ) and weight fraction (*w*) for **C0–Na**, **C3–Na**, and **C3–Ca** (fitting lines are shown as a guide). (c) Photographs of corresponding dish-cast films (cast suspensions: 2.7 mL with [CNC] = 1.5 wt % and [NaCl] = 1.5 mM ) taken on a dark background. Scale bar 2.0 cm.

The anisotropic phase formed by the CNCs displays a left-handed “cholesteric” structure, also commonly called “chiral nematic”, whereby the elongated constituents point locally parallel to one another in a direction that twists helicoidally into a left-handed periodic structure. The distance over which the local orientation of the CNCs completes a full rotation about the helical axis is called the pitch (*p*, in μm). The pitch, which is related to the chiral interactions between the CNCs, is known to decrease as Φ increases.^38,39^ For cotton CNCs, *p* is found to be inversely proportional to Φ, which implies that the strength of the chiral interactions between neighboring CNCs can be measured through a parameter defined as the chiral strength (κ, in μm^–1^, and defined as positive for simplicity) expressed as^40^

3 $$\frac{2\pi }{p}=\kappa \Phi$$

The evolution of the pitch as a function of the CNC volume fraction in Figure 7b reveals that **C3–Na** had a smaller pitch than **C0–Na**. The corresponding chiral strengths obtained by fitting eq 3 are 26 ± 1 μm^–1^ for **C0–Na** and 44 ± 2 μm^–1^ for **C3–Na**, suggesting a stronger chiral interaction for the centrifuged CNCs. These observations are in agreement with the previously reported positive correlation between chiral strength and bundle proportion in the CNC population.^8^ These results suggest that the centrifugation step leading to **C3**, and commonly applied in most laboratory-made CNCs, is responsible for the formation of additional bundles that increase the effective left-handed chiral strength of the suspension and decrease the cholesteric pitch.

The ability of CNCs to self-organize into cholesteric structures can be harnessed to make structurally colored films. Such a film is produced by dish-casting a CNC suspension, leading to the formation of a cholesteric structure of decreasing pitch upon slow drying into a solid film. When the pitch in the resulting film is comparable to the wavelength (λ) of visible light, a selective reflection occurs for λ = *n p*, where *n* is the average refractive index of cellulose. The reflected wavelength λ is thus proportional to the pitch *p* in the film, and from eq 3, it is also proportional to 1/κ.^8,41,42^ Consequently, an increase of the chiral strength of the CNCs is expected to cause a blue-shift of the film color. To illustrate the practical significance of this behavior, the suspensions were used to produce such CNC films, as presented in Figure 7c. The samples **C0–Na** and **C3–Na** both formed structurally colored films, with the **C0–Na** film exhibiting a green center with a red edge while the **C3–Na** exhibited a blue center with a greenish edge. This shows that using centrifugation as a purification step causes a significant blue-shift of the subsequent CNC film and thus is of significant practical importance for applications that exploit the chiral self-assembly properties of CNCs.

The interpretation of the evolution of the measured anisotropic volume fraction and pitch for **C3–Ca** is less straightforward. This sample exhibited a steep increase of φ_*ani*_ with increasing CNC volume fraction Φ (Figure 7a), which is usually indicative of early gelation.^43^ Between crossed polarizers, this sample displayed distinctive shear-alignment above 6 wt % (Figure S16), indicating that the sample was kinetically arrested and was not able to relax over time, leading to an apparent φ_*ani*_ close to 100%. Such a gelation at a low CNC volume fraction can be caused by the greater hydrodynamic volume induced by the irregular composite particles. Despite these clear signs of gelation, **C3–Ca** exhibited some fingerprint patterns at intermediate Φ arising from isolated tactoids, suggesting that local cholesteric ordering occurred in the transient stage between sample preparation and gelation.^2^ In these regions, the evolution of *p* as a function of Φ followed the same trend as for **C3–Na**. This suggests that calcium-induced aggregation did not increase the chiral strength of the CNCs, but that a subpopulation of chiral bundles is still present. The coexistence of subpopulations of irregular composite particles and of chiral bundles is consistent with the broadening of the size distribution toward larger sizes while maintaining a significant proportion of small sizes (see Figure 4c,d). Nevertheless, the film made from **C3–Ca** appeared colorless and slightly hazy (Figure 4e), which we can ascribe to early gelation promoted by the irregular particles. The behavior of this sample illustrates the critical difference between the two types of composite particles.

More generally, these results highlight the practical consequences of CNC suspension history for their self-organization behavior. To illustrate this point, the self-assembly of CNC samples with different bundle content and ionic strength history was investigated. The results, presented in Figure S17, demonstrate that transient exposure to an increased ionic strength (NaCl) by concentration followed by dilution during capillary preparation leads to an increase of particle size and a convergence of their self-assembly behavior (see discussion in Section S8 for more details).

### High-Shear Viscosity of the CNC Suspensions

The characterization of the morphological properties and of the self-organization behavior of the different samples suggests that the different types of composite particles exhibit different hydrodynamic behaviors, with potential importance for their use as rheological modifiers. Moreover, the rheological properties of diluted suspensions can provide indirect information about the particle morphology and are also exempt from drying artifact and statistical noise. For these reasons, the evolution of the relative viscosity (η_*r*_) of the samples was investigated as a function of the particle concentration *c* (g mL^–1^), as reported in Figure 8. Both **C0–Na** and **C3–Na** displayed a similar evolution of the viscosity with the concentration, in accordance with previous studies.^44^ However, despite exhibiting earlier gelation, the relative viscosity of **C3–Ca** was always lower than for **C0–Na** and **C3–Na**. This result indicates that **C3–Ca** exhibits a greater dependence of the viscosity with the CNC concentration and/or the shear rate than **C0–Na** and **C3–Na**.^45,46^

**Figure 8:**
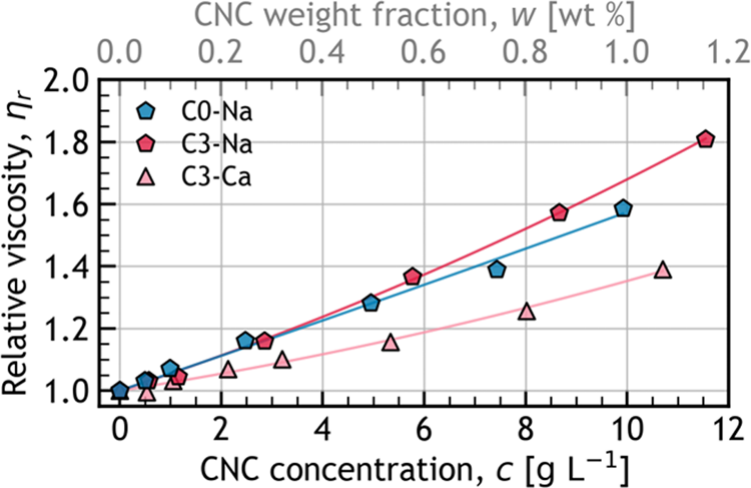
Plot of the relative viscosity (η_r_) as a function of the CNC concentration (*c*) with best fit using eq 4.

The viscometry measurements were used to extract the intrinsic viscosity [η] (mL g^–1^) and the dimensionless Huggins coefficient (*k*_H_) using the Huggins equation (see Table S15):^47^

4 $$\eta_{r}\approx 1+\left[\eta \right]c+k_{H}{[\eta ]}^{2}c^{2}$$

This analysis revealed that **C3–Na** and **C0–Na** exhibited similar intrinsic viscosities (54 ± 2 mL g^–1^ and 56 ± 6 mL g^–1^, respectively) that were greater than that of **C3–Ca** (25 ± 3 mL g^–1^). This trend is in agreement with a decrease of the aspect ratio of the particles.^48^ Assuming the CNCs can be modeled as prolate ellipsoids, the intrinsic viscosity can be used to extract their shape factor *r*, defined as their 3D aspect ratio, according to^49,50^

5 $$\left[\eta \right]=\frac{8}{15}\frac{\left(r^{4}-1\right)}{\rho_{\mathrm{cell}}r^{2}\left[\frac{\left(2r^{2}-1\right)\cosh^{-1}\left(r\right)}{r\sqrt{r^{2}-1}}-1\right]}$$

where *ρ_cell_* is the CNC volumetric mass density (taken to be 1.6 g mL^–1^).^51^ Both **C3–Na** and **C0–Na** had the same shape factor (*r* = 35), which was greater than the shape factor exhibited by **C3–Ca** (*r* = 22) (see Table S15). These results indicate a similarity of 3D aspect ratio between never-centrifuged and centrifuged CNCs, while highlighting a lower aspect ratio for the Ca-CNCs, in accordance with the other morphological analyses. The values of shape factors obtained by viscometry are significantly larger than the aspect ratios obtained from the analysis of TEM images. This discrepancy can be explained by the presence of electroviscous effects that cause a significant increase in apparent aspect ratio when the measurements are performed without salt addition,^52,53^ which was the case for this study.

The Huggins coefficients (*k*_*H*_), which appear as second-order additive correction factors in eq 4 and characterize particle interactions, were extracted and reported in Table S15. Both **C3–Na** and **C0–Na** exhibited similarly low *k*_*H*_ values (0.5 ± 0.1 and 0.1 ± 0.2, respectively), which are both distinctly smaller than for **C3–Ca** (1.6 ± 0.8). All were in the range previously reported for CNCs,^54^ and for other comparable charged elongated nanoparticles.^48^ The significantly greater *k*_*H*_ displayed by **C3–Ca** indicates that Ca-CNCs displayed much stronger mutual interactions (either repulsive or attractive). This is consistent with the lower absolute value of the ζ-potential of Ca-CNCs (ζ ≈ −35 mV) compared to that of pH-neutralized CNCs (ζ ≈ −43 mV) (see Table S1). Together, the greater *k*_*H*_ and lower ζ-potential of Ca-CNCs are indicative of a lower colloidal stability of the Ca-CNCs, in agreement with the formation of bigger particles and the adsorption of tightly bound calcium cations on the particle surface.^55^

In conclusion, calcium-induced aggregation of CNCs can be of interest for rheological applications where a high viscosity or gel-like behavior is desired at rest. However, this increase in viscosity comes at the cost of reduced colloidal stability. Overall, the rheological properties of Ca-CNCs could be investigated further to assess their dynamic and concentration-dependent behavior.

### Fragmentation of CNC Aggregates

Mild ultrasonication (*e.g*. using a bath sonicator) is often used to redisperse loose CNC aggregates, with a minor impact on the morphology of the crystallites.^2,56^ However, significantly increasing the ultrasonication dose eventually leads to near-complete fragmentation of the composite particles back into individual crystallites.^8,57^ Consequently, ultrasonication is accompanied by a drastic reduction of the Z-average diameter of the CNCs.^8,24,58,59^

According to DLVO theory, irregular composite CNCs are less stable than laterally aligned CNCs.^28^ Therefore, randomly associated composite particles should be easier to fragment than the laterally associated bundles. To explore this, the different CNC suspensions were exposed to increasing ultrasonication dose (*u*, J mL^–1^) and the Z-average diameter (*D*_*H*_) was monitored by DLS (Figure 9). For all samples, *D*_*H*_ decreased sharply with increasing *u*, as previously observed for CNCs.^8,24,58,59^ Eventually, all samples reached a similar plateau of *D*_*H*_^*∞*^ = 59 ± 2 nm for *u* > 445 J mL^–1^, despite having different initial sizes. At low dose (*u* < 30 J mL^–1^), the samples showed two different behaviors with **C3** and **C0** displaying a moderate size change (∼5 nm mL J^–1^) while **C3–Ca** and **C0–Ca** exhibit a greater size drop (∼18 nm mL J^–1^).

**Figure 9:**
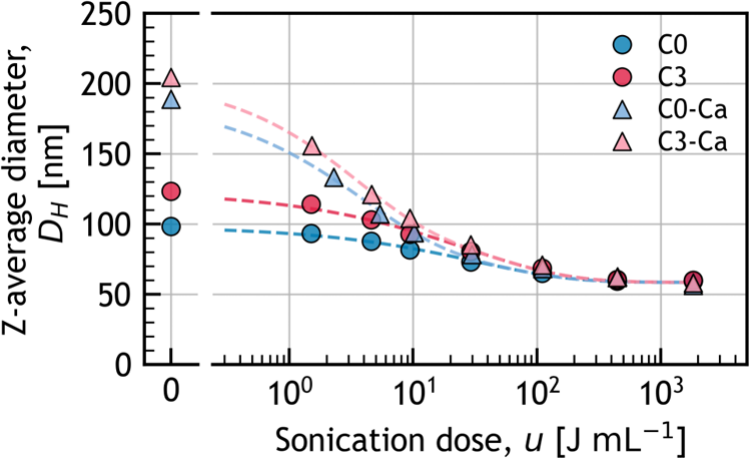
Impact of ultrasonication dose (*u*) on the Z-average diameter (*D*_H_) fitted using eqs S24 and S25 (dashed line, see Section S10 for more details).

The evolution of the particle size as a function of particle type and sonication dose was also modeled by using a modified dissociation expression (Figure 9 see Section S10 for more details). This analysis further shows that the ultrasonication dose has a greater impact on the size change of calcium-induced composite particles than it has on bundles (see Table S16).

This result suggests that the calcium-induced irregular composite particles are less tightly bound than the bundle-like particles. This is in accordance with the higher energetic configuration and weaker energetic barrier predicted by DLVO theory for cross-associated CNCs compared to laterally associated CNCs.^28^ This analysis shows that both irregular composite particles and bundle objects are broken down by prolonged ultrasonication and thus should be avoided when the presence of composite particles is desired.

## Conclusions

In this study, we explored the impact of centrifugation and transient exposure to high ionic strength on the properties of CNCs, through a thorough characterization of their individual morphologies, internal structures, and collective chiral properties in suspensions. The impact of these treatments was quantified after extensive dialysis against ultrapure water, and compared to the control suspension using DLS, zetametry, TEM, SAXS, SNBED, viscometry, and other experimental comparative techniques.

We showed that the first centrifugation step applied in most CNC extraction processes at the laboratory scale is responsible for the formation of additional CNC “bundles” with raft-like morphology. These raft-like particles stemmed from the preferential lateral association of crystallites through their hydrophobic faces, which occurred without loss of the number of exposed sulfate half-ester groups. Despite their lower proportion of hydrophobic surfaces, the bundles did not significantly modify the surfactant ability of the suspension. However, the bundles enhanced the chiral strength of the suspension, leading to a clear blue-shift of the corresponding, structurally colored, dish-cast film. This step could be further explored and adjusted via the centrifugation force, duration, and ionic environment to provide further control on the CNC chirality.

An alternative aggregation pathway was explored through the destabilization of purified CNC induced by CaCl_2_ addition followed by dialysis against ultrapure water. The corresponding CNCs were in the form of calcium salts and exhibited a clear increase of the proportion of irregular composite particles as indicated by their increased average size, irregular morphology, lower 3D aspect ratio, and irregular internal structure. Consequently, these particles exhibited a smaller ζ-potential and promoted gelation at lower volume fraction while exhibiting a lower viscosity at high shear. As demonstrated in this study, this aggregation pathway can be adjusted with salt concentration and type, with potential applications as rheological modifiers.

This work raises broader questions about both the origin of bundles and the chiral character of CNCs. Cellulose fibers often display a chiral arrangement in the plant cell wall,^60^ and drying treatments of the cellulose source prior to hydrolysis can cause irreversible fiber aggregation (*i.e.*, hornification).^61^ As a result, it has often been suggested that chiral CNC bundles are inherited from these preexisting structures in the plant tissue. By showing that exposure to high ionic strength during centrifugation favors the formation of bundles that enhance chirality, this work implies that the bundles initially present in never-centrifuged suspensions could also originate from the same mechanism during the hydrolysis and the quenching steps, since these conditions can also trigger irreversible aggregation of the cellulose crystallites (when they assemble through their hydrophobic faces). Consequently, centrifugation at high ionic strength may not create a new type of chiral-enhancing bundles but rather amplifies their formation. Quantitative investigations using alternative cellulosic sources (*e.g*. never-dried starting material), pretreatment (*e.g*. hornification) or hydrolysis conditions (*e.g*. temperature, acid) could be of interest to assess the origin and chiral properties of the bundles present in never-centrifuged suspensions.

To conclude, our results highlight how overlooked variations in the CNC extraction protocol can be critical in terms of suspension behavior. Importantly, while these findings are based on the analysis of CNCs obtained from sulfuric acid hydrolysis of cotton, they are likely applicable to different sources and methods. This encourages further investigation of CNCs extracted from wood or obtained by other production methods, which also typically display a dominant proportion of bundled particles.^6,62–64^ These results are especially important to consider when transferring findings from laboratory-made to industrial CNCs, as the latter are typically purified by ultrafiltration instead of centrifugation,^65^ which is expected to impact their particle morphology and colloidal properties. As such, this work pinpoints previously unexplored possibilities with immediate practical considerations for commercial applications of CNCs, where their chiral liquid crystalline properties or their amphiphilicity are directly exploited, but also in any other situation where the CNC morphology and surface chemistry are key.

## Methods

### Materials

Sulfuric acid (H_2_SO_4_, ≥95%, analytical grade), sodium hydroxide (NaOH, 99%, pellets), sodium chloride (NaCl, ≥99.5%, laboratory grade), calcium chloride (CaCl_2_, fused granular) and hydrogen peroxide (H_2_O_2_, > 30 w/v%, laboratory reagent grade) were provided by Fisher Scientific. Hexadecane (Sigma-Aldrich reagent plus 99%) was passed through basic silica before use. All water used in this work was type 1 ultrapure water (Milli-Q, Millipore, Synergy UV system).

**Data processing** was performed with custom-made Python scripts. Statistical analyses and data fitting were performed with the Scipy and LMFIT libraries, respectively.

**CNCs suspensions (C0–C3)** were produced by sulfuric acid hydrolysis of cotton-derived filter paper (Whatman No. 1). Shredded filter paper (15 g, CookWork coffee grinder) was introduced in a 63.9 ± 0.1 wt % sulfuric acid solution (300 g, ρ = 1.543 ± 0.001 g mL^–1^, hydrometer ISO 650, 1.500–1.600, Scientific Laboratory Supplies) preheated to 60 °C. After 30 min of hydrolysis under vigorous mechanical stirring, the medium was quenched with ice-cold water (300 mL). Part of the mixture was set aside, and the rest was centrifuged (20,000 *g*, 20 min, 4 °C, Lynx 6000 Thermo Scientific, T29-8 × 50 rotor), with the resulting pellet redispersed in water. This process was repeated to produce aliquots of CNCs that had never been centrifuged (**C0**), centrifuged once (**C1**), twice (**C2**), and three times (**C3)**. All samples were then dialyzed against deionized water (MWCO 12–14 kDa, Medicell membrane), with the water changed at least once a day until the conductivity was stable (around 2 weeks). The suspensions were passed through cellulose nitrate filters (8 then 0.8 μm, Sartorius)

**CNC mass fraction** was obtained from gravimetric analysis by drying the suspensions in an oven (65 °C, > 40 h). The mass of dry CNC was at least 15 mg, measurements were made in triplicate.

**CNC surface sulfate half-ester groups** were quantified by conductometric titration of CNC suspensions diluted in water (approximately 180 mL) with the addition of NaCl solution (0.1 M, 2 mL). An automatic titrator (Metrohm, 800 Dosino) was used to inject NaOH solution (10 mM Titripur, 50 μL min^–1^) while monitoring the conductivity (856 conductivity module). The number of CNC surface sulfate half-ester groups was deduced from the first equivalence point.

**Concentrated CNC suspensions (C0–Na, C3–Na)** were prepared by neutralizing the suspensions with 1 molar equiv of NaOH per CNC surface sulfate half-ester group followed by concentrating with a rotavapor (35 °C, 20 mbar).

**Salt-induced irreversible aggregates** were prepared by diluting concentrated CNC suspension in water before pipetting CaCl_2_ or NaCl aqueous solution to form a 2 – 6.5 wt % CNC suspension in an ionic strength of 0 to 80 mM. The mixture was centrifuged (10,000 g for 20 min, Minispin Eppendorf), redispersed, then diluted for Zetasizer Measurements measurement as described below. The ionic strength of the CNCs due to their surface ions was neglected.

**Dialyzed calcium-aggregated CNC suspensions (C0–Ca, C3–Ca)** were obtained by preparing 6.5 wt % CNC suspensions of C0–Na or C3–Na containing CaCl_2_ (13.2 ± 0.2 mM), followed by centrifugation (10,000*g* for 20 min), redispersion (to approximately 1 wt % CNC), and dialysis against water (≥2 weeks). For self-assembly measurements, C3–Ca was first concentrated with a rotavapor (35 °C, 20 mbar), then further concentrated by evaporation under ambient conditions.

**The calcium content** was measured by inductively coupled plasma-optical emission spectrometry (ICP-OES, Thermo Fisher Scientific iCAP 7400 Duo ICP Spectrometer). Freeze-dried CNCs (∼20 mg) were further dried overnight in an oven (60 °C). The precisely weighed CNCs were digested for 1 h in freshly prepared piranha solution (3:1 v/v H_2_SO_4_:H_2_O_2_). A known amount of the mixture was diluted in water to obtain a solution of (approximately 3.6 wt % acid, approximately 2 g L^–1^ CNCs) that was used for the measurement. ICP Standard (Sigma-Aldrich) were diluted with approximately 2% nitric acid (TraceMetal grade, Fisher) in water (TraceSelect for Trace Analysis, Honeywell Riedel-de Haen) to make the standard curve. Analysis was performed using Qtegra software.

**Zetasizer measurements** were performed on dilute CNC suspensions (0.1 wt %), and NaCl was used to set the ionic strength as close as possible to 1 mM. The Z-average hydrodynamic diameters were estimated in backscattering geometry (173°, 633 nm, Malvern Zetasizer Nano ZS) from three runs of ten measurements after an initial waiting time of 5 min for the temperature to equilibrate (20–22 °C). The zeta potential was acquired after the Z-average measurement through three runs of ten measurements and analyzed using the Smoluchowski equation. Data are presented as mean ± standard deviation.

**TEM** was performed with a Talos F200X G2 microscope (FEI, 200 kV, CCD camera). A drop of CNC suspension (0.002 wt %, in 1 mM NaCl) was deposited on a glow-discharged carbon-coated copper grid. After 2 min, the excess solution was blotted with filter paper. Then, a drop of uranyl acetate aqueous solution (2 wt %) was deposited and let to sit for 1.5 min before blotting again. Particles (*N* ≥ 225) were manually outlined using Fiji (ImageJ), with all touching objects considered as a discrete CNC particle. Outlines were processed using the Shape Filter plugin to extract the bounding box length (*L*_*b*_), width (*W*_*b*_), and the particle area (*A*).^66^ The bounding box aspect ratio (*r*_*b*_) and rectangularity (*Rect*) were calculated from eqs 1 and 2 respectively. Values were compared using a Mann–Whitney *U* test and the magnitude of differences between samples was quantified using the point biserial correlation coefficient (for more details, see Section S4.2).

**Cryo-TEM** was performed using a JEOL JEM 2100Plus (Jeol, Japan), operated at 200 kV, equipped with a Gatan RIO 16 camera (Gatan Inc., U.S.A.). Cryo-frozen samples were prepared using an EM GP2 Automatic Plunge Freezer (Leica Microsystems, Germany) to vitrify the sample by rapid immersion in liquid ethane. The images presented were contrast-enhanced to highlight the CNCs.

**SAXS measurements** were performed at the ID02 beamline of European Synchrotron Radiation Facility (ESRF) using X-rays of 12.23 keV. Two-dimensional X-ray scattering patterns were recorded on an Eiger2 4 M pixel detector (Dectris) at a detector distance of *ca.* 10 m. The CNC suspensions were sealed in glass capillaries of an outer diameter of 1 mm and wall thickness of 0.13 mm. The obtained 2D data were then azimuthally averaged using SAXSutilities.^67^

**SNBED** data were acquired in a low-dose condition optimized for cellulose crystals as described previously, using a JEOL 2100F operating at 200 kV equipped with a NanoMEGAS ASTAR system. The nanobeam configuration consisted of a converged electron probe of 25 nm. An ED pattern was recorded at every probe position using a Cheetah Medipix3 direct electron detector (manufactured by Amsterdam Scientific Instruments) with a 0.5 ms exposure time per probe position. The diffraction data sets were analyzed using a dedicated ASTAR software to perform: (i) crystal orientation identification through correlation with templates (*i.e.*, precomputed theoretical patterns) and (ii) Virtual–Bright (VBF) and Virtual–Dark field (VDF) image reconstruction that consists of plotting the intensity fluctuations of the transmitted beam (VBF) and user-selected diffraction positions (VDF) over the scanned area.

**Selected area ED experiments** were carried out with a JEM-2100Plus TEM operated at 200 kV using a selected area aperture of a diameter of 1 μm. The CNC suspensions were deposited on a glow-discharged carbon-coated copper grid. The excess liquid was removed by filter-paper blotting, then the grids were dried in air. Two-dimensional ED patterns were recorded from areas of CNCs with local orientation along the fiber axis on a MerlinEM hybrid pixel detector (Quantum Detectors) with an exposure time of 10 fps in a continuous acquisition mode. Equatorial ED profiles were obtained using the first 10 consecutive frames of the recorded data sets using an in-house program.

**Interfacial tension** between CNC suspensions (0.9 wt %) in 20 mM NaCl and hexadecane was measured through the pendant drop method with a FTA1000 Analyzer System (with a 22 gauge needle). For each sample, four photographs per drop (approximately 35 μL) were taken every minute starting 1 min after its formation, for at least six drops (*N* = 6 × 4 pictures). The surface tension (γ) was calculated for each picture through the method of a selected plane. The in house software developed for this purpose is available on Github: DropPyTension (see Section S6 for more details).^68,69^ The corresponding Bond numbers were in [0.16, 0.22] and the apparent Worthington numbers in [0.66, 0.93], indicating that the measurement conditions were satisfactory to extract γ with accuracy.^70^

**Liquid crystalline properties** were investigated by observation of CNC suspensions in glass capillaries. The flat capillaries (CM Scientific, ID = 0.3 × 6.0 mm^2^) were filled with a series of suspension dilutions prior to sealing with nail varnish and marking the initial meniscus position (so that any evaporation could be accounted for). The anisotropic volume fraction and corrected concentration were measured from the analysis of images taken after 2 weeks and again after at least a further week to confirm that no further evolution occurred. The pitch was measured after 2 weeks using polarized optical microscopy. Images were recorded in brightfield transmission configuration using a Zeiss Axio microscope in Koehler illumination equipped with a 50x objective (Nikon T Plan SLWD, NA 0.4) and a CMOS camera (UI- 3580LE-C-HQ, IDS). At least four images were recorded at different locations in the anisotropic phase, with three pitch measurements performed per image (*N* ≥ 12).

**Structurally colored films** were made by slowly drying 2.7 mL of 1.5 wt % CNC suspension with 1.5 mM of NaCl in polystyrene Petri dishes (35 mm diameter). The pictures were taken against a black background using a camera oriented at approximately 30° from the normal of the film that was under diffuse illumination.

**Relative viscosity** was calculated from flow time measurements at low particle concentration by using

6 $$\eta_{r}=\frac{t\rho }{t_{0}\rho_{0}}\approx \frac{t}{t_{0}}$$

where *t* and *t_0_* are the flow times of the suspension and of the solvent respectively and *ρ* and *ρ_0_* are the densities of the suspension and the solvent, respectively. Flow times were measured in triplicate with an Ubbelohde viscometer (Technico, 0.05 cSt s^–1^) in air at 20 °C. The flow time of water was estimated to be 18.65 ± 0.09 s (measured five times in triplicate, *N* = 15). The intrinsic viscosity, and the Huggins coefficient, were extracted by fitting the relative viscosity as a function of the CNC concentration according to eq 4 and the 3D aspect ratio of the samples was estimated from the intrinsic viscosity by using eq 5. The choice of approach and equation derivation are described in more detail in Section S9.1.

**Ultrasonication** of dilute CNC solutions (30 – 40 mL, 0.1 wt % CNCs, 1 mM NaCl) was performed in an ice bath using a Fisherbrand Ultrasonic disintegrator (20 kHz, ø = 12.7 mm, pulses 2:1 s On:Off, 40% amplitude). Samples were ultrasonicated for regular intervals of increasing time in between which aliquots were removed for analysis (1 mL). The dose received by each sample (J mL^–1^) was calculated for each step from ultrasonication time divided by the volume of the sample and multiplied by the true power delivered by the probe to the sample (20 W, determined by calorimetry by Parton et al.^8,71^

## Supplementary Material



## Data Availability

The data that support the findings of this study are openly available in the University of Cambridge data repository at 10.17863/CAM.110504.

## Notes

DLS-derived count rate, additional aggregation experiments, TEM distributions and statistics, Cryo-TEM images, SAXS analysis details, SNBED illustration and discussion, SAED, interfacial tension experiments, capillary images, additional self-assembly experiments, viscometry analysis, details on the fit of Z-average diameter as a function of sonication (PDF)
