# Supplementary material for: Tailoring the Morphology of Cellulose Nanocrystals via Controlled Aggregation
Source: ACS Nano. 2025 Jul 2;19(27):25228–42. doi: 10.1021/acsnano.5c05548 (PMC12269359; doi:10.1021/acsnano.5c05548)
Supplement: Supplementary file 1 [file nn5c05548_si_001.pdf]

SUPPORTING INFORMATION

Tailoring the Morphology of Cellulose Nanocrystals via Controlled Aggregation

Kévin Ballu[a], Jia-Hui Lim[b], Thomas G. Parton[c], Richard M. Parker[a], Bruno Frka-Petesic[a,d], Alexei A. Lapkin[e,f], Yu Ogawa[b], Silvia Vignolini[a,c]**

[a]Yusuf Hamied Department of Chemistry, University of Cambridge, Cambridge CB2 1EW, United Kingdom

[b]University of Grenoble Alpes, CNRS, CERMAV, 38000 Grenoble, France

[c]Department of Sustainable and Bio-inspired Materials, Max Planck Institute of Colloids and Interfaces, 14476 Potsdam, Germany

[d]International Institute for Sustainability with Knotted Chiral Meta Matter (WPI-SKCM^2), Hiroshima University, Hiroshima 739-8526, Japan

[e]Department of Chemical Engineering and Biotechnology, University of Cambridge, Cambridge CB3 0AS, United Kingdom

[f]Innovative Center in Digital Molecular Technologies, Yusuf Hamied Department of Chemistry, University of Cambridge, Cambridge CB2 1EW, United Kingdom

Table of Content

S1. CNC Hydrodynamic and Surface Properties 3

S1.1 CNC Dynamic Light Scattering (DLS) Derived Count Rate 4

S2. Salt-induced CNC aggregation 6

S2.1 Influence of process parameters on salt-induced CNC aggregation 6

S2.2 Influence of salt type and ionic strength on salt-induced CNC aggregation 8

S3. TEM and Cryo-TEM analyses 10

S3.1 Histograms of TEM Values 10

S3.2 Distribution of the TEM Values 12

S4.2 Statistical Analysis of TEM Values 16

S3.3 Cryo-TEM images 21

S4. SAXS 22

S4.1 SAXS Profile Modeling Expression 22

S4.2 Thickness Extraction 23

S4.3 Cross-sectional Guinier Analysis 24

S5. Electron Diffraction Analyses 25

S5.1 Typical Scanning Nanobeam Electron Diffraction (SNBED) Data 25

S5.2 Impact of Favored Interaction Between the Crystallites and the Grid 26

S5.3 Selected Area Electron Diffraction (SAED) 27

S6. Interfacial Tension Measurements 28

S6.1 Extraction of Interfacial Tension from Drop Images 28

S6.2 Interfacial Tension Measurement of CNC Suspensions 29

S7. Capillary images of C3-Ca 31

S8 Impact of Salt Condition on CNC Self-Organization 32

S9. Viscometry 34

S9.1 Fitting of the Relative Viscosity 34

S9.2 Expression for 3D Aspect Ratio 36

S10. Fitting the Z-average Diameter as a Function of Ultrasonication Dose 37

S11. References 39

S1. CNC Hydrodynamic and Surface Properties

Table S1. Summary of the CNC characteristics: Z-average diameter (D_H), ζ-potential, surface sulfate half-ester groups ($-OSO_3^-$) per CNC mass from titration measurements, corresponding surface charge per surface area, and calcium content from elemental analysis. The surface charge per surface area was calculated by considering spherical CNCs with a diameter equal to their measured Z-average diameter.

Sample	D_H *[nm]*	ζ-potential [mV]	Sulfate half-ester [mmol kg^{-1}]	Surface charge [e nm^{-2}]	Ca content *[*mmol kg^{-1}]
**C0**	99 ± 1	-48 ± 5	264 ± 5	4.2 ± 0.1	N/A
**C1**	129 ± 1	-50 ± 8	269 ± 5	5.6 ± 0.1	N/A
**C2**	127 ± 1	-49 ± 5	267 ± 5	5.5 ± 0.1	N/A
**C3**	124 ± 2	-50 ± 8	264 ± 5	5.3 ± 0.1	N/A
**C0-Na**	94 ± 1	-41 ± 4	N/A	N/A	N/A
**C3-Na**	114 ± 1	-44 ± 1	N/A	N/A	6
**C0-Ca**	189 ± 1	-35 ± 1	N/A	N/A	N/A
**C3-Ca**	216 ± 9	-35 ± 2	N/A	N/A	133

S1.1 CNC Dynamic Light Scattering (DLS) Derived Count Rate

For a diluted suspension of scattering particles (in the single-scattering regime), the scattered light intensity measured in a fixed solid angle segment $\mathrm{d}\Omega = \sin(\theta)\,\mathrm{d}\theta\,\mathrm{d}\varphi$ is proportional to the product of the differential scattering cross-section of the particles ($\mathrm{d}\sigma_s/\mathrm{d}\Omega$) and their number density (n_s):

$$I(\theta,\varphi) \propto \frac{\mathrm{d}\sigma_s}{\mathrm{d}\Omega}\, n_s \tag{S1}$$

For comparing CNC samples at equal mass concentration γ_s (g L^{-1}) the scattering intensity is proportional to the differential cross-section per scatterer mass:

$$I(\theta,\varphi) \propto \frac{\mathrm{d}\sigma_s}{\mathrm{d}\Omega}\frac{\gamma_s}{m_s} \tag{S2}$$

When performing a dynamic light scattering (DLS) measurement with a Malvern Zetasizer, it is possible to access the derived count rate (DCR) in kilocounts per second (kcps) which is directly proportional to the scattering intensity (multiplied by a device-dependent constant c),[1] and thus to the differential scattering cross section:

$$DCR = c\,I \propto \frac{\mathrm{d}\sigma_s}{\mathrm{d}\Omega}\, n_s \tag{S3}$$

The scattering cross-section of a particle depends on its size, shape and optical contrast with the surrounding medium. Consequently, comparison of the DCR with the Z-average hydrodynamic diameter of different samples, measured at similar concentration, can give an indication of the relative compactness of the particles.

Figure S1 shows values for the DCR of the samples versus an effective "hydrodynamic volume" given by the Z-average diameter cubed (D_H^3). All samples were measured at similar CNC mass concentration (0.1 wt%) and should therefore have identical volume fraction but varying number density. All the non-Ca samples could be placed on a single line DCR $= m\,D_H^3$, whereas for the Ca-CNCs samples the increase of DCR per D_H^3 is comparatively smaller. This result suggests that the effective density of the **C0**, **C1**, **C2** and **C3** samples (*i.e.* mass enclosed within its effective hydrodynamic volume) is similar, while the Ca-CNCs have a significantly lower effective density. This finding is consistent with the hypothesis that centrifugation-induced aggregation leads to the formation of compact aligned particles while the calcium-induced aggregation yields randomly associated particles.

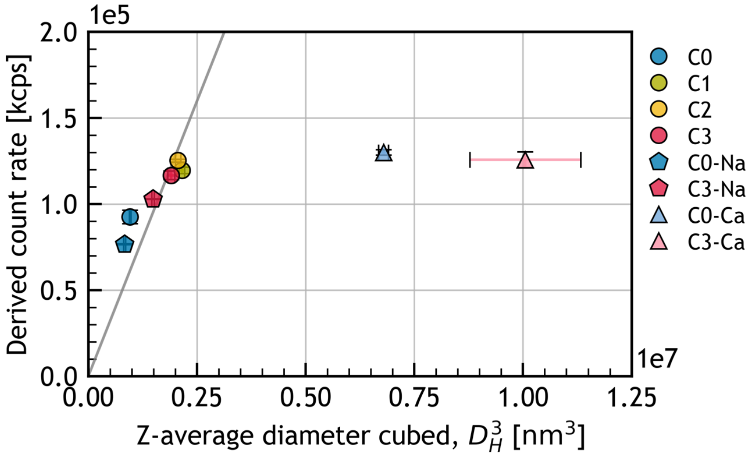

Figure S1. Evolution of the dynamic light scattering (DLS) derived count rate with the Z-average diameter cubed (D_H^3) for different CNC samples measured at similar concentration (0.1 wt% CNCs). The linear fitting line is presented as a guide to the eye.

S2. Salt-induced CNC aggregation

S2.1 Influence of process parameters on salt-induced CNC aggregation

The impact of process parameters during the salt-induced aggregation of CNCs was investigated by DLS measurements. A CNC suspension of **C3-Na** was prepared with added $CaCl_2$ to reach a transiently raised ionic strength I_{agg} = 51 mM and a CNC weight fraction of w_{agg} = 6.5 wt%. After a given time (0 h to 5 days) or centrifugation treatment (one or two 20 min cycles at 10,000 g), the suspension was redispersed and diluted in deionized water to a stable CNC suspension with w_{DLS} = 0.1 wt% and at an ionic strength I_{DLS} = 1 mM. The Z-average diameter (D_H) of the resulting particles measured by DLS are presented in **Table S2**. In these conditions, the waiting time before redispersion and dilution seemed to have a negligible impact beyond 1 h. Similarly, applying any number of centrifugation cycles led to similar particle sizes, indicating that this does not influence the size of the particles after dilution. Consequently, the aggregation step of the salt-induced aggregation experiments was carried out using one centrifugation cycle.

Table S2. Impact of the aggregation time (t_{agg}) and number of centrifugation cycles (N_{centri}) on the Z-average diameter (D_H) of diluted CNCs after salt-induced aggregation of **C3-Na** at w_{agg} = 6.5 wt% CNC and I_{agg} = 51 mM of ionic strength induced by $CaCl_2$ addition.

t_{agg}	N_{centri}	D_H [nm]
0 h		209 ± 1
1 h		293 ± 2
3 h	0	299 ± 4
24 h		270 ± 2
196 h		275 ± 3
N/A	1	285 ± 5
	2	274 ± 2
50 days	1	274 ± 6

The impact of the CNC weight fraction during aggregation (w_{agg}) at fixed transiently raised ionic strength (I_{agg}) was also investigated. **C3-Na** suspensions over a range of CNC weight fraction ($2.0 \leq w_{agg} \leq 6.5$ wt%) were centrifuged at I_{agg} = 36 mM, followed by redispersion and dilution (w_{DLS} = 0.1 wt%, I_{DLS} = 1 mM) before measuring their D_H. As shown in **Figure S2**, lowering the CNC weight fraction led to an increase of D_H after aggregation from 243 to 311 nm. This effect could be due to the decrease of viscosity, facilitating the formation of contact points between the particles. Alternatively, it is possible that at higher weight fraction, CNC are better packed, leading to denser aggregates. Therefore, the next aggregation experiments, presented in **Figure 3b**, were all carried out with a fixed CNC weight fraction during aggregation of w_{agg} = 6.5 wt%.

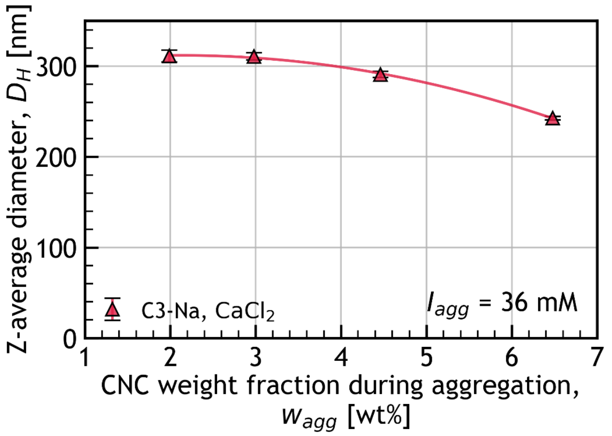

Figure S2. Evolution of the Z-average diameter (D_H) as a function of the CNC (**C3-Na**) mass fraction (w_{agg}) during calcium aggregation at fixed ionic strength (I_{agg} = 36 mM). The line of best fit, presented as a visual guide, was obtained from a second order polynomial.

S2.2 Influence of salt type and ionic strength on salt-induced CNC aggregation

The aggregation protocol presented in **Figure 3a** was followed to investigate the impact of the salt-type and ionic strength on the properties of the CNC suspensions after centrifugation and on their D_H after dilution. We compared the impact of NaCl and $CaCl_2$ at various transiently raised ionic strengths ($0 \leq I_{agg} \leq 80$ mM) for both **C0-Na** and **C3-Na** ($w_{agg} = 6.5$ wt%) on the properties of the suspension in two steps. First, after the aggregation step (i.e. after salt addition and centrifugation), leading to biphasic systems made up of a bottom birefringent arrested phase, and an upper clear liquid layer. Then, after redispersion and dilution to a stable CNC suspension with $w_{DLS} = 0.1$ wt% and at an ionic strength (I_{DLS}) as close as possible to 1 mM.

After the aggregation step, the bottom phase of the CNC suspension was qualitatively inspected. For both salts, increasing I_{agg} from 0 to 80 mM led to gelation of the bottom layer, that required an increasing effort to be redispersed. The volume of the gel layer decreased with increasing I_{agg}, indicating an increase of the density of the arrested phase. Overall, these qualitative inspections indicate that for all salt types and CNC samples, increasing the ionic strength led to the formation of a stronger gel.

In the second step, the obtained CNC suspensions were redispersed, diluted and their D_H was measured. The time between sample centrifugation and their dilution for measurement varied from 0 to 63 days without significant impact on the D_H trend, as presented in **Figure S3**. The evolution of D_H was highly dependent on the salt type and transiently raised ionic strength, as illustrated in **Figure 3b**.

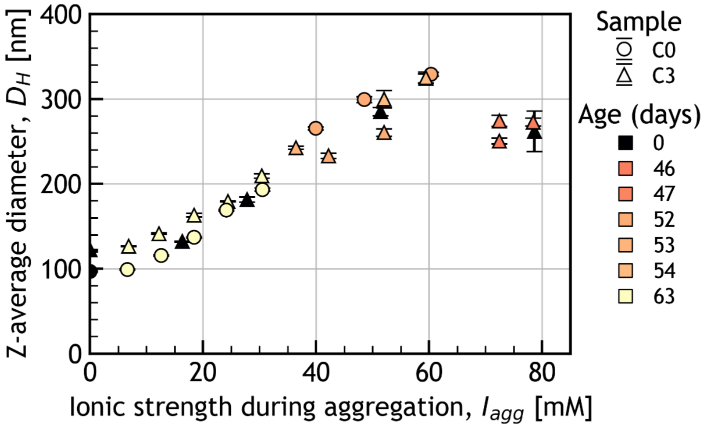

Figure S3. Evolution of the CNC Z-average size (D_H) after dilution following calcium-induced aggregation as a function of ionic strength (I_{agg}), CNC sample, and waiting time before dilution.

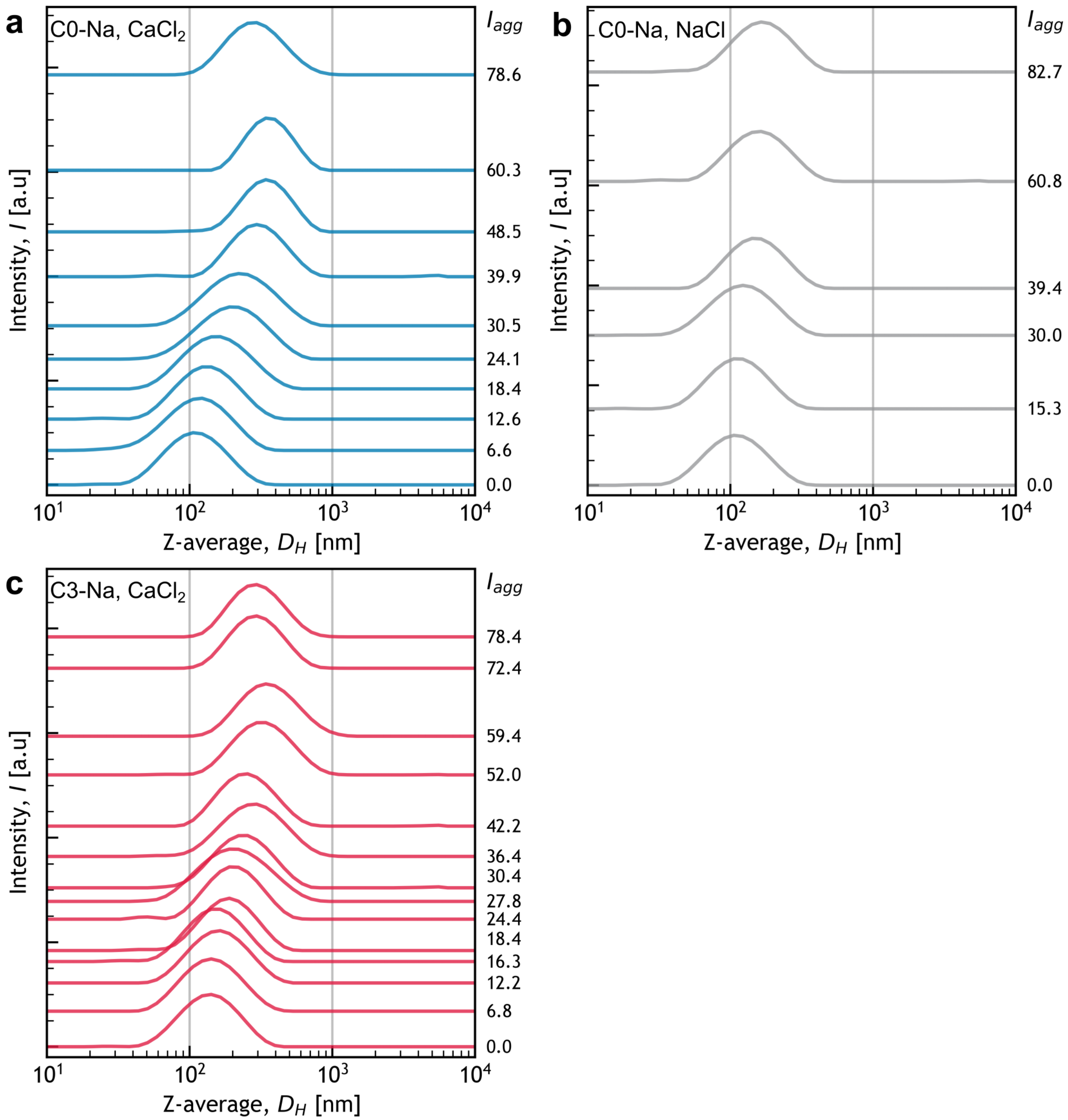

Figure S4. Evolution of the DLS scattered intensity (I_{abs}) as a function of the Z-average diameter (D_H) after dilution following salt-induced aggregation as a function of the transiently raised ionic strength (I_{agg}) for **(a) C0-Na** with NaCl, **(b) C0-Na** with $CaCl_2$ and **(c) C3-Na** with $CaCl_2$. Measurements were performed after redispersion and dilution to w_{DLS} = 0.1 wt% and an ionic strength (I_{DLS}) as close as possible to 1 mM.

S3. TEM and Cryo-TEM analyses

S3.1 Histograms of TEM Values

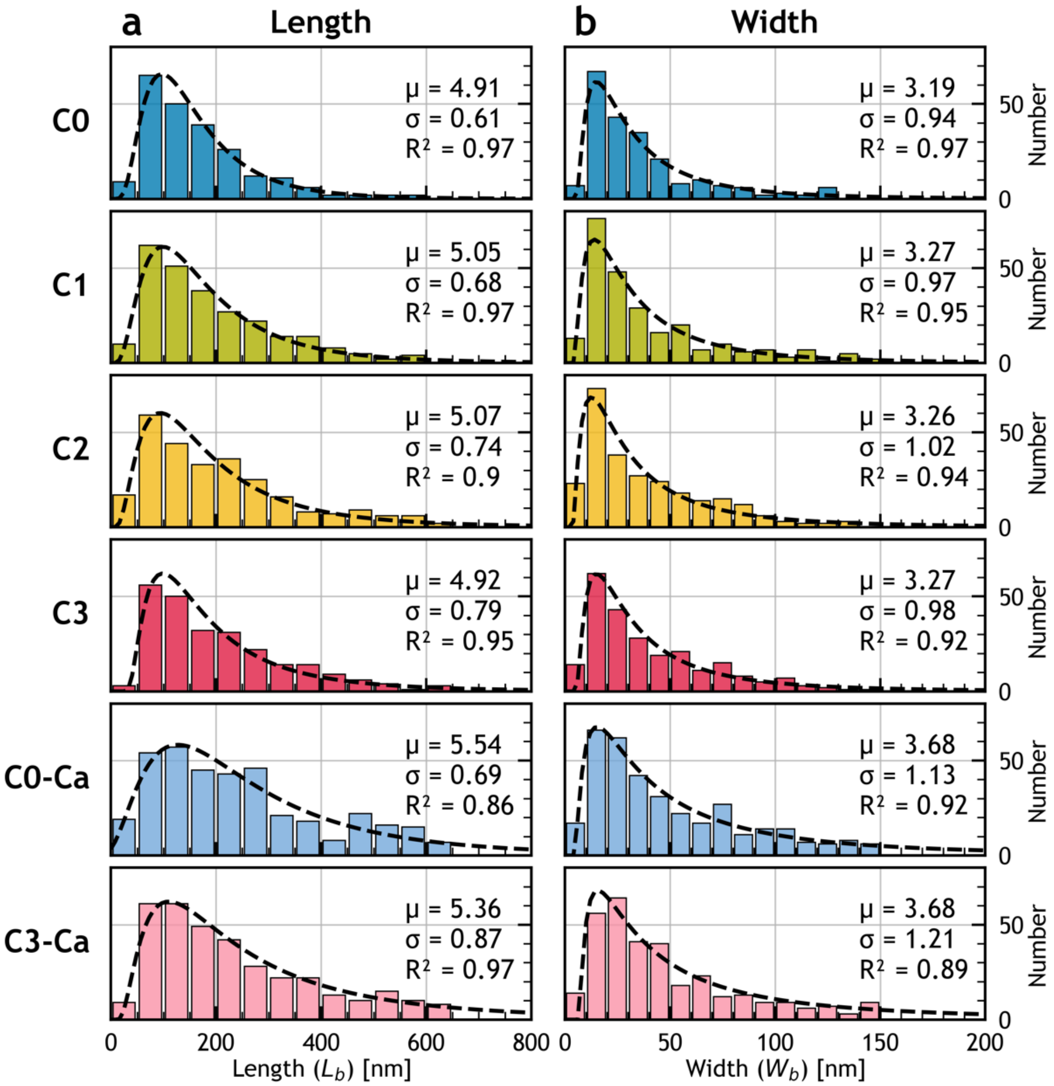

Figure S5. Histograms of CNC dimensions from analysis of TEM images (**a**) bounding box length (L_b) and (**b**) bounding box width (W_b). The data were fitted with a log-normal distribution and the corresponding fitting parameters are presented for each plot.

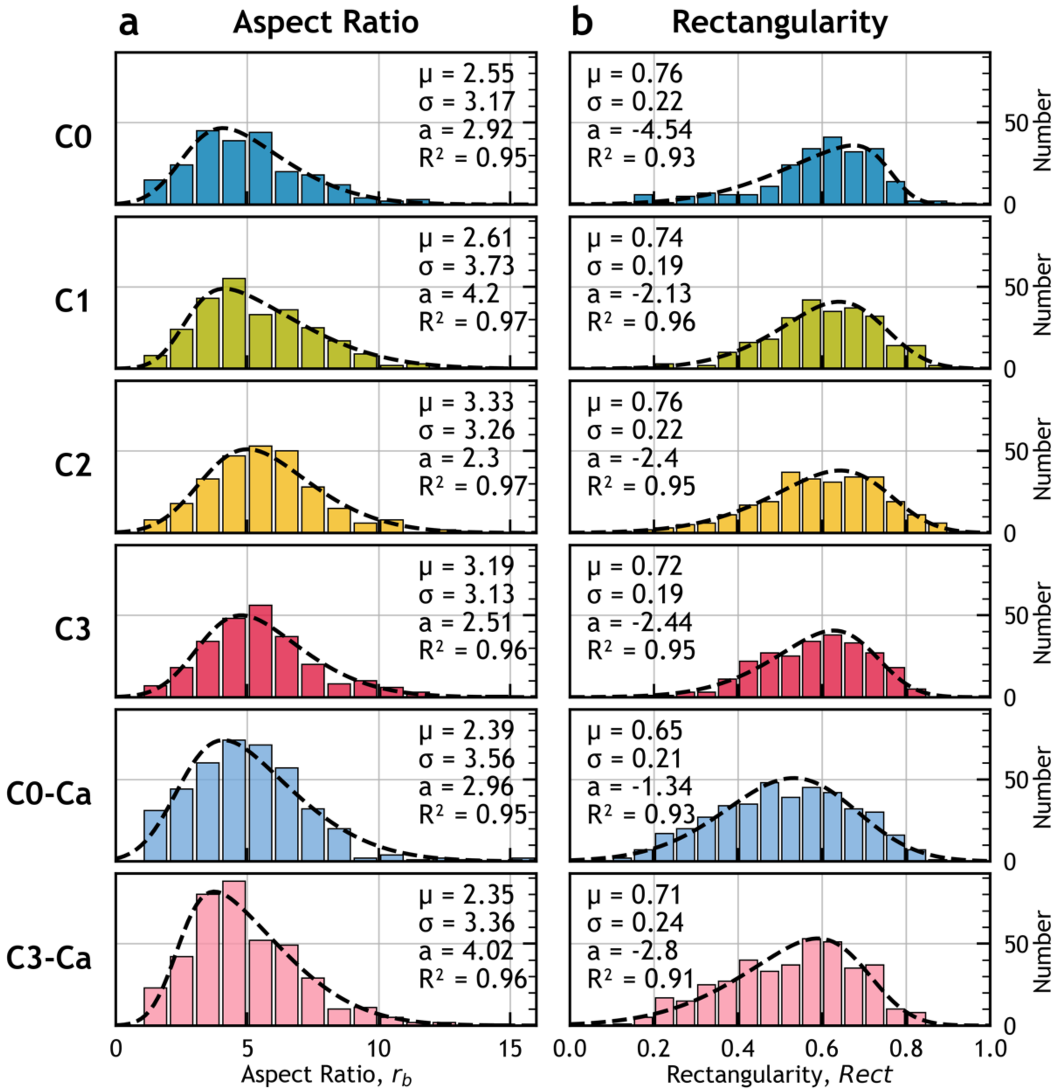

Figure S6. Histograms of CNC shape parameters from analysis of TEM images (**a**) bounding box aspect ratio (r_b) and (**b**) rectangularity (*Rect*). The data were fitted by skewed-normal distributions, the corresponding fitting parameters are presented for each plot.

S3.2 Distribution of the TEM Values

The values obtained by TEM for the bounding box lengths (L_b) and bounding box widths (W_b) seem to follow a log-normal distribution (see **Figure S5**). If that is the case, then ln (L_b) and ln (W_b) should follow a normal distribution. The normality of a distribution can be investigated graphically through a normal quantile-quantile (Q-Q) plot, showing the quantiles of the investigated distribution against the quantiles of a normal distribution. If the points follow a perfectly linear evolution, then the investigated distribution follow a normal distribution. **Figure S7** shows the normal Q-Q plot for ln (L_b) and ln (W_b), for all the samples most of the data followed a linear evolution with small deviations at low and high quantiles indicating that the underlying distributions are close to normal.

Graphical investigations of normality are often used in conjunction with analytical tests such as the Shapiro–Wilk test. This test compares the distribution of a dataset to a normal distribution through calculation of the statistic W, $\in [0;1]$, for which a value of one indicates normality of the data, and a corresponding *p*-value below the chosen confidence level indicates that there is evidence that the dataset is not normally distributed.[2] As presented in **Table S3**, for ln (L_b) and ln (W_b) most samples display some deviation from normality when using a confidence threshold of 0.05.

The same investigations were applied to the corresponding bounding box aspect ratios (r_b) and rectangularities (*Rect*) that seemed to follow a skewed normal distribution (see **Figure S6**). The normal Q-Q plots for r_b and *Rect* presented in **Figure S8** exhibit some constant deviation from normality and all the corresponding Shapiro–Wilk test indicate non-normality (**Table S3**). More precisely r_b and *Rect* values displayed a right and left skew respectively.

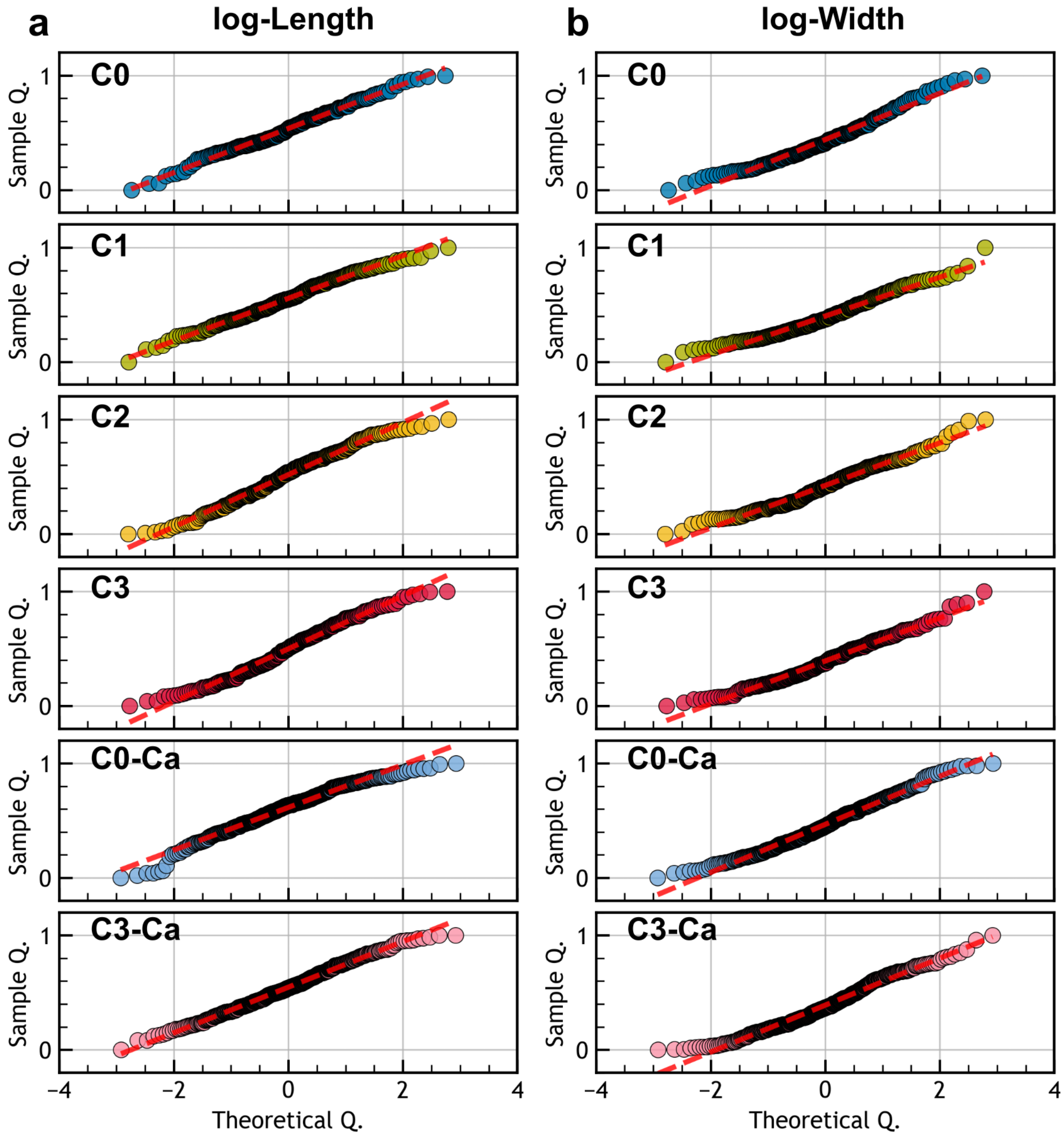

Figure S7. Normal quantile-quantile (Q-Q) plots of the CNC shape parameters from analysis of TEM images (**a**) logarithm of bounding box length, ln (L_b), and (**b**) logarithm of bounding box width, ln (W_b).

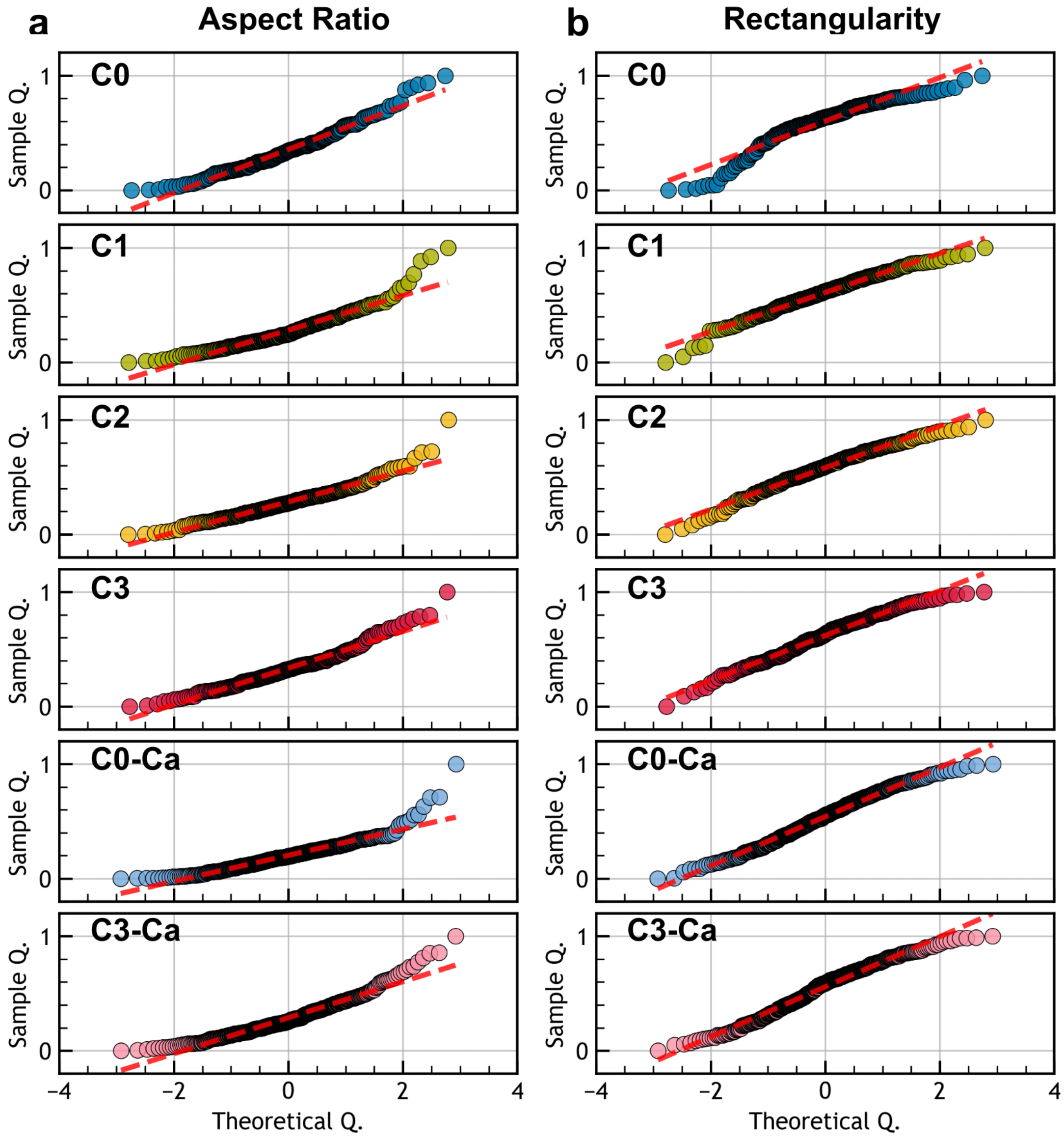

Figure S8. Normal quantile-quantile (Q-Q) plots of the CNC shape parameters from analysis of TEM images (**a**) bounding box aspect ratio (r_b) and (**b**) rectangularity (*Rect*).

Table S3. Shapiro–Wilk test of normality *W* statistic and corresponding *p*-values for the logarithm of the bounding box lengths (L_b) and widths (W_b) and for the aspect ratio (r_b) and rectangularity (*Rect*) for each sample (*i.e.* the *p*-value can be viewed as the probability to obtain these specific sample distributions if the corresponding population was normally distributed). The distribution of the data highlighted in red does not differ significantly from a normal distribution with a confidence threshold of 0.05.

Sample	$\ln(L_b)$	$\ln(W_b)$	r_b	*Rect*
**C0**	0.993<br>(0.346)	0.976<br>(0.001)	0.971<br>(0.000)	0.922<br>(0.000)
**C1**	0.992<br>(0.155)	0.977<br>(0.000)	0.933<br>(0.000)	0.983<br>(0.004)
**C2**	0.984<br>(0.005)	0.982<br>(0.002)	0.958<br>(0.000)	0.983<br>(0.003)
**C3**	0.982<br>(0.003)	0.984<br>(0.007)	0.967<br>(0.000)	0.984<br>(0.008)
**C0-Ca**	0.978<br>(0.000)	0.987<br>(0.001)	0.921<br>(0.000)	0.987<br>(0.001)
**C3-Ca**	0.993<br>(0.087)	0.980<br>(0.000)	0.952<br>(0.000)	0.979<br>(0.000)

S4.2 Statistical Analysis of TEM Values

The statistical significance of the differences of average values between samples can be estimated through different tests. The most common test for such a comparison is the Student t-test. This test requires the underlying populations to be normally distributed, which in our case is uncertain. Consequently, pairwise comparison between samples was performed using a Mann–Whitney *U* test, as it does not assume normality, does not necessitate any transformation of the data and is generally more powerful.[3] Note that the corresponding Student t-tests were also performed for comparison, leading to equivalent results (see **Table S5**, **Table S7**, **Table S9**, and **Table S11**).

The size of the effects was also estimated through calculation of the point biserial correlation coefficient, r, according to:[4]

$$r = \frac{z}{\sqrt{N}} \tag{S4}$$

where N is the pooled sample size and z is the normalized U statistics calculated from:

$$z = \frac{U - 0.5 n_1 n_2}{\sqrt{\frac{n_1 n_2 (n_1 + n_2 + 1)}{12}}} \tag{S5}$$

with n_1 and n_2 the sample sizes. The results of the Mann–Whitney U test and the corresponding r for L_b, W_b, r_b and *Rect* are presented respectively in **Table S4**, **Table S6**, **Table S8**, and **Table S10**, and summarized in **Figure 4**.

Table S4. Mann–Whitney *U* statistics, corresponding *p*-value and point biserial correlation coefficient resulting from the pairwise comparison of L_b for all samples. Data highlighted in red indicates that the corresponding samples do not differ significantly with a confidence threshold of 0.05.

	C0	C1	C2	C3	C0-Ca	C3-Ca
**C0**	/	32384 (0.043) **-0.09**	34667 (0.009) **-0.12**	32849 (0.001) **-0.15**	60965 (0.000) **-0.28**	60493 (0.000) **-0.30**
**C1**	32384 (0.043) **0.09**	/	36022 (0.548) -0.03	34029 (0.246) -0.05	64522 (0.000) **-0.20**	64121 (0.000) **-0.22**
**C2**	34667 (0.009) **0.12**	36022 (0.548) 0.03	/	32521 (0.573) -0.02	43106 (0.000) **-0.17**	41304 (0.000) **-0.19**
**C3**	32849 (0.001) **0.15**	34029 (0.246) 0.05	32521 (0.573) 0.02	/	59359 (0.000) **-0.16**	38783 (0.000) **-0.17**
**C0-Ca**	60965 (0.000) **0.28**	64522 (0.000) **0.20**	43106 (0.000) **0.17**	59359 (0.000) **0.16**	/	77495 (0.601) -0.02
**C3-Ca**	60493 (0.000) **0.30**	64121 (0.000) **0.22**	41304 (0.000) **0.19**	38783 (0.000) **0.17**	77495 (0.601) 0.02	/

Table S5. Absolute Student-t statistics and corresponding *p*-value resulting from the pairwise comparison of ln (L_b) for all samples. Data highlighted in red indicates that the corresponding samples do not differ significantly with a confidence threshold of 0.05.

	C0	C1	C2	C3	C0-Ca	C3-Ca
**C0**	/	2.05 (0.041)	2.53 (0.012)	3.51 (0.000)	7.28 (0.000)	8.56 (0.000)
**C1**	2.05 (0.041)	/	0.52 (0.603)	1.38 (0.167)	5.05 (0.000)	6.27 (0.000)
**C2**	2.53 (0.012)	0.52 (0.603)	/	0.82 (0.411)	4.39 (0.000)	5.57 (0.000)
**C3**	3.51 (0.000)	1.38 (0.167)	0.82 (0.411)	/	3.71 (0.000)	4.94 (0.000)
**C0-Ca**	7.28 (0.000)	5.05 (0.000)	4.39 (0.000)	3.71 (0.000)	/	1.19 (0.234)
**C3-Ca**	8.56 (0.000)	6.27 (0.000)	5.57 (0.000)	4.94 (0.000)	1.19 (0.234)	/

Table S6. Mann–Whitney *U* statistics, corresponding *p*-value and point biserial correlation coefficient resulting from the pairwise comparison of W_b for all samples. Data highlighted in red indicates that the corresponding samples do not differ significantly with a confidence threshold of 0.05.

	C0	C1	C2	C3	C0-Ca	C3-Ca
**C0**	/	29398 (0.932)<br>-0.00	30499 (0.995)<br>0.00	29804 (0.232)<br>-0.05	56773 (0.000)<br>**-0.21**	57477 (0.000)<br>**-0.24**
**C1**	29398 (0.932)<br>0.00	/	34637 (0.852)<br>0.01	33844 (0.295)<br>-0.05	64491 (0.000)<br>**-0.20**	65297 (0.000)<br>**-0.24**
**C2**	30499 (0.995)<br>-0.00	34637 (0.852)<br>-0.01	/	31518 (0.249)<br>-0.05	41250 (0.000)<br>**-0.20**	38551 (0.000)<br>**-0.23**
**C3**	29804 (0.232)<br>0.05	33844 (0.295)<br>0.05	31518 (0.249)<br>0.05	/	59734 (0.000)<br>**-0.17**	37217 (0.000)<br>**-0.20**
**C0-Ca**	56773 (0.000)<br>**0.21**	64491 (0.000)<br>**0.20**	41250 (0.000)<br>**0.20**	59734 (0.000)<br>**0.17**	/	76159 (0.349)<br>-0.03
**C3-Ca**	57477 (0.000)<br>**0.24**	65297 (0.000)<br>**0.24**	38551 (0.000)<br>**0.23**	37217 (0.000)<br>**0.2**	76159 (0.349)<br>0.03	/

Table S7. Absolute Student-t statistics and corresponding *p*-value resulting from the pairwise comparison of ln (W_b) for all samples. Data highlighted in red indicates that the corresponding samples do not differ significantly with a confidence threshold of 0.05.

	C0	C1	C2	C3	C0-Ca	C3-Ca
**C0**	/	0.21 (0.835)	0.01 (0.989)	1.09 (0.277)	5.90 (0.000)	6.94 (0.000)
**C1**	0.21 (0.835)	/	0.22 (0.829)	0.87 (0.385)	5.63 (0.000)	6.66 (0.000)
**C2**	0.01 (0.989)	0.22 (0.829)	/	1.08 (0.283)	5.76 (0.000)	6.78 (0.000)
**C3**	1.09 (0.277)	0.87 (0.385)	1.08 (0.283)	/	4.80 (0.000)	5.85 (0.000)
**C0-Ca**	5.90 (0.000)	5.63 (0.000)	5.76 (0.000)	4.80 (0.000)	/	1.09 (0.278)
**C3-Ca**	6.94 (0.000)	6.66 (0.000)	6.78 (0.000)	5.85 (0.000)	1.09 (0.278)	/

Table S8. Mann–Whitney *U* statistics, corresponding *p*-value and point biserial correlation coefficient resulting from the pairwise comparison of r_b for all samples. Data highlighted in red indicates that the corresponding samples do not differ significantly with a confidence threshold of 0.05.

	C0	C1	C2	C3	C0-Ca	C3-Ca
**C0**	/	32692 (0.026) **-0.10**	36685 (0.000) **-0.17**	32436 (0.003) **-0.14**	46531 (0.613) -0.02	43841 (0.751) 0.01
**C1**	32692 (0.026) **0.10**	/	37759 (0.112) -0.07	33140 (0.535) -0.03	47209 (0.043) **0.08**	44161 (0.004) **0.11**
**C2**	36685 (0.000) **0.17**	37759 (0.112) 0.07	/	35444 (0.249) 0.05	63658 (0.000) **0.15**	64770 (0.000) **0.19**
**C3**	32436 (0.003) **0.14**	33140 (0.535) 0.03	35444 (0.249) -0.05	/	43810 (0.009) **0.10**	57202 (0.000) **0.14**
**C0-Ca**	46531 (0.613) 0.02	47209 (0.043) **-0.08**	63658 (0.000) **-0.15**	43810 (0.009) **-0.10**	/	82397 (0.323) 0.04
**C3-Ca**	43841 (0.751) -0.01	44161 (0.004) **-0.11**	64770 (0.000) **-0.19**	57202 (0.000) **-0.14**	82397 (0.323) -0.04	/

Table S9. Absolute Student-t statistics and corresponding *p*-value resulting from the pairwise comparison of r_b for all samples. Data highlighted in red indicates that the corresponding samples do not differ significantly with a confidence threshold of 0.05.

	C0	C1	C2	C3	C0-Ca	C3-Ca
**C0**	/	2.59 (0.010)	3.8 (0.000)	2.84 (0.005)	0.6 (0.547)	0.08 (0.936)
**C1**	2.59 (0.010)	/	1.06 (0.289)	0.12 (0.906)	2.19 (0.029)	2.94 (0.003)
**C2**	3.8 (0.000)	1.06 (0.289)	/	0.99 (0.321)	3.49 (0.001)	4.33 (0.000)
**C3**	2.84 (0.005)	0.12 (0.906)	0.99 (0.321)	/	2.45 (0.014)	3.26 (0.001)
**C0-Ca**	0.6 (0.547)	2.19 (0.029)	3.49 (0.001)	2.45 (0.014)	/	0.77 (0.440)
**C3-Ca**	0.08 (0.936)	2.94 (0.003)	4.33 (0.000)	3.26 (0.001)	0.77 (0.440)	/

Table S10. Mann–Whitney *U* statistics, corresponding *p*-value and point biserial correlation coefficient resulting from the pairwise comparison of *Rect* for all samples. Data highlighted in red indicates that the corresponding samples do not differ significantly with a confidence threshold of 0.05.

	C0	C1	C2	C3	C0-Ca	C3-Ca
**C0**	/	29523 (0.868)<br>-0.01	29961 (0.730)<br>0.02	25770 (0.130)<br>0.07	31253 (0.000)<br>**0.26**	32523 (0.000)<br>**0.22**
**C1**	29523 (0.868)<br>0.01	/	33933 (0.557)<br>0.03	29224 (0.080)<br>0.08	34806 (0.000)<br>**0.28**	36449 (0.000)<br>**0.24**
**C2**	29961 (0.730)<br>-0.02	33933 (0.557)<br>-0.03	/	35385 (0.263)<br>0.05	70043 (0.000)<br>**0.25**	66313 (0.000)<br>**0.21**
**C3**	25770 (0.130)<br>-0.07	29224 (0.080)<br>-0.08	35385 (0.263)<br>-0.05	/	36859 (0.000)<br>**0.22**	59164 (0.000)<br>**0.18**
**C0-Ca**	31253 (0.000)<br>**-0.26**	34806 (0.000)<br>**-0.28**	70043 (0.000)<br>**-0.25**	36859 (0.000)<br>**-0.22**	/	75276 (0.227)<br>-0.04
**C3-Ca**	32523 (0.000)<br>**-0.22**	36449 (0.000)<br>**-0.24**	66313 (0.000)<br>**-0.21**	59164 (0.000)<br>**-0.18**	75276 (0.227)<br>0.04	/

Table S11. Absolute Student-t statistics and corresponding *p*-value resulting from the pairwise comparison of *Rect* for all samples. Data highlighted in red indicates that the corresponding samples do not differ significantly with a confidence threshold of 0.05.

	C0	C1	C2	C3	C0-Ca	C3-Ca
**C0**	/	0.89 (0.374)	0.08 (0.938)	0.71 (0.475)	6.45 (0.000)	5.47 (0.000)
**C1**	0.89 (0.374)	/	0.83 (0.405)	1.75 (0.081)	7.98 (0.000)	6.94 (0.000)
**C2**	0.08 (0.938)	0.83 (0.405)	/	0.82 (0.413)	6.73 (0.000)	5.72 (0.000)
**C3**	0.71 (0.475)	1.75 (0.081)	0.82 (0.413)	/	6.27 (0.000)	5.20 (0.000)
**C0-Ca**	6.45 (0.000)	7.98 (0.000)	6.73 (0.000)	6.27 (0.000)	/	1.14 (0.256)
**C3-Ca**	5.47 (0.000)	6.94 (0.000)	5.72 (0.000)	5.20 (0.000)	1.14 (0.256)	/

S3.3 Cryo-TEM images

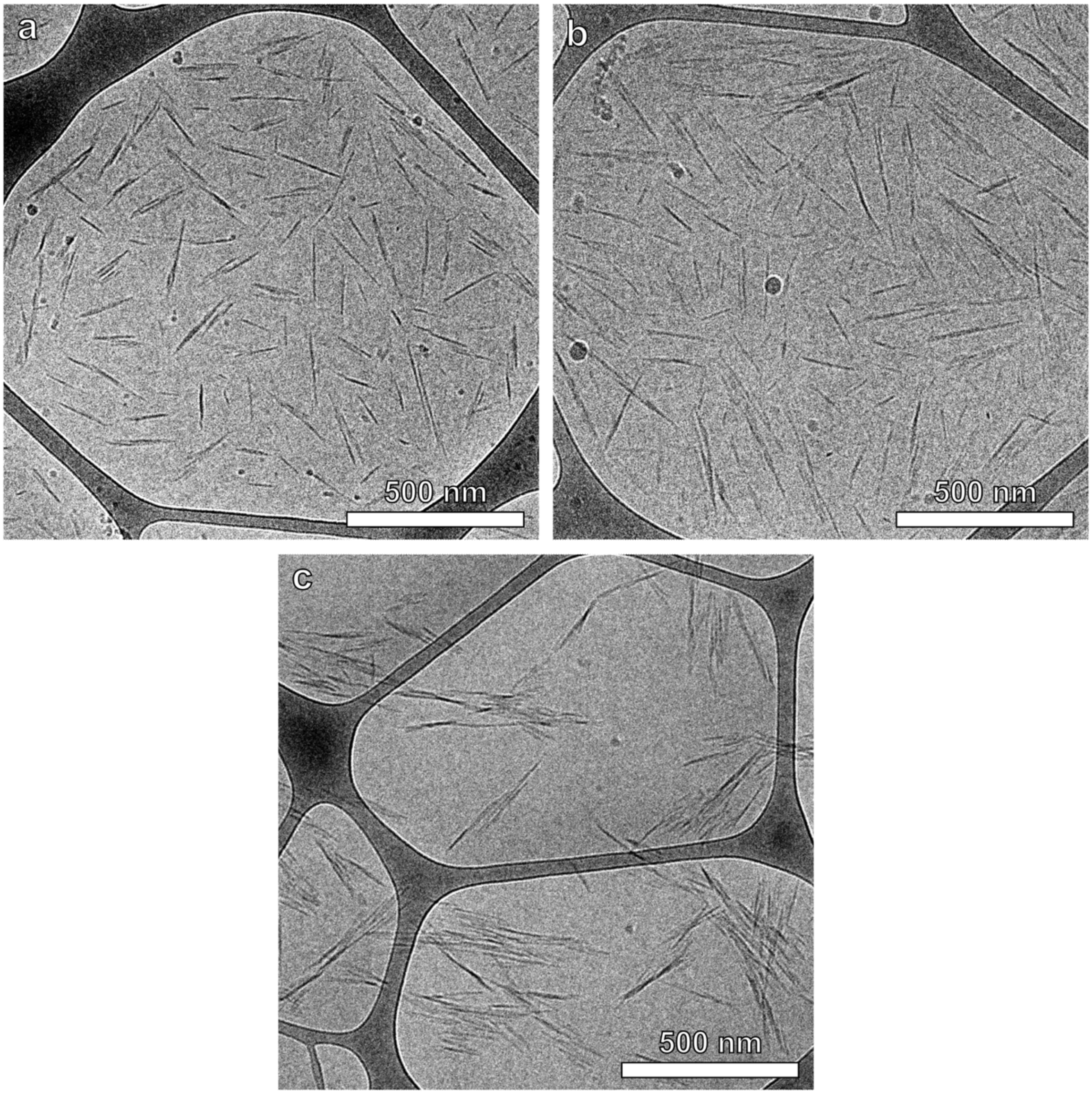

Figure S9. Typical images from Cryo-TEM of (**a**) never centrifuged CNCs (**C0**), (**b**) triply-centrifuged CNCs (**C3**) and (**c**) Ca-aggregated CNCs (**C3-Ca**).

S4. SAXS

S4.1 SAXS Profile Modeling Expression

At low particle concentration, the SAXS intensity profile of a monodisperse sample, $I(q)$ (cm^{-1}), can be expressed in terms of the orientationally averaged form factor $P(q)$ as:[5]

$$I(q) = \Phi \, \Delta\rho^2 P(q) V_p^2 \tag{S6}$$

where Φ (v/v%) is the particle volume fraction, V_p (cm^3) is the volume the particles and $\Delta\rho$ (cm^{-2}) is the scattering contrast, given by:[6]

$$\Delta\rho = \left(\frac{n_{e,0}\rho_{cell}}{M_0} N_A - \tilde{\rho}_{solv}\right) r_0 \tag{S7}$$

with $n_{e,0}$ and M_0 are respectively the number of electron and the molar mass of a repeating unit (86 e and 162.1 g mol^{-1} respectively), ρ_{cell} is the cellulose mass density (taken as 1.6 g cm^{-3} for a Iβ cellulose crystal),[7] N_A is the Avogadro constant (6.02 10^{23} mol^{-1}), $\tilde{\boldsymbol{\rho}}_{\boldsymbol{solv}}$ is the electronic number density of water (3.34 10^{23} e cm^{-3}),[6] and r_0 is the scattering length of an electron (2.82 10^{-13} cm).[6]

For polydisperse systems, the orientationally averaged form factor contribution from each particle $P_i(q)$ must be accounted for. The total intensity then becomes:

$$I(q) = \Phi \, \Delta\rho^2 \frac{1}{N\langle V\rangle} \sum_{i=1}^{N} P_i(q) V_i^2 \tag{S8}$$

where N is the number of particles, V_i the volume of particle i., and $\langle V\rangle$ is the average particle volume. The orientationally averaged form factor for each particle $P_i(q)$ can be estimated by averaging the form factor F_i of the particle over several orientations (M), as expressed by:

$$P_i(q) = \frac{1}{M} \sum_{j=1}^{M} \left|F_i(q, \theta_j, \varphi_j)\right|^2 \tag{S9}$$

where the form factor of a rectangular prism of length L_i, width W_i and thickness T_i is given by:[5,6]

$$F_i(q,\theta,\varphi) = \mathrm{sinc}\left(\frac{L_i\, q \cos(\theta)}{2}\right) \mathrm{sinc}\left(\frac{W_i\, q \sin(\theta)\sin(\varphi)}{2}\right) \mathrm{sinc}\left(\frac{T_i\, q \sin(\theta)\cos(\varphi)}{2}\right) \tag{S10}$$

with $\mathrm{sinc}(x) = \sin(x)/x$.

For each sample, the SAXS profile at $c = 0.1$ wt % was resampled to 100 q-values evenly spaced in logarithmic scale. For each q, 800 particles were generated with their length and width randomly drawn from the distribution of L_b and W_b (extracted from TEM), and with a fixed thickness (T). The orientationally averaged form factor $P_i(q)$ for each particle was computed over 200 random orientations using **Equation** S9. The total SAXS intensity at each q was then obtained by averaging over the 800 particles according to **Equation** S8.

S4.2 Thickness Extraction

Using the method above, we modeled for each sample their corresponding SAXS profile over a range of thickness values *T* to extract the best fitting thickness. In practice, we first evaluate for each sample the coefficient of determination R^2 between the experimental and modeled profiles for each *T* value (**Figure S10**). Then, we fit the dependence of R^2 with *T* using a second order polynomial function. This allows for the extraction of the best thickness (T_b) corresponding to the optimum value of *T* that maximizes R^2. The resulting SAXS profiles, modeled for each sample at their respective T_b, are presented in **Figure 5c**.

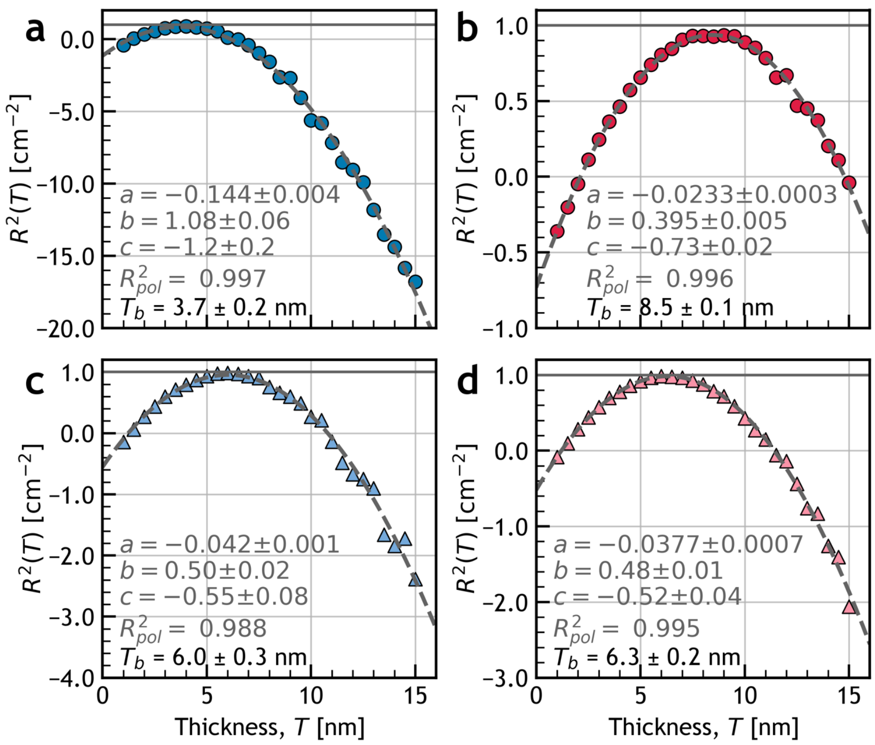

Figure S10: Evolution of the coefficient of determination (R^2) between the experimental SAXS profiles and the unoptimized modeled profiles over a range of thickness values (*T*). Best fit obtained from a second order polynomial function $R^2 = aT^2+bT+c$ (dashed black line), corresponding parameters, coefficient of determination (R_{pol}^2), and best-fit thickness (T_b) indicated. The horizontal grey line indicates R^2 = 1.

S4.3 Cross-sectional Guinier Analysis

Similarly, for elongated rods at intermediate q, the scattered intensity can be expressed as:[6,8]

$$I(q) = \frac{1}{q}\pi c \frac{\Delta\rho^2}{\rho_{cell}^2} m_L \exp\left(-\frac{q^2 R_C^2}{2}\right), \qquad \frac{2\pi}{L} < q < \frac{1}{R_c} \tag{S11}$$

where m_L (g cm^{-1}) is the particle mass per unit length and R_c (cm) is the cross-sectional gyration radius. This analysis was performed for all the samples (see **Figure S11**) and the corresponding m_L and R_c are presented in **Figure 5c**.

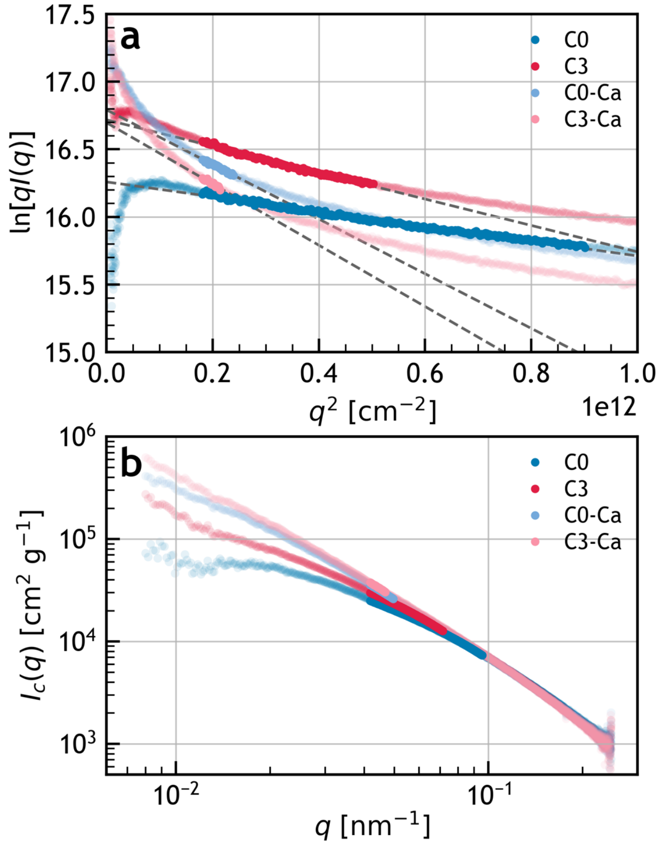

Figure S11. Overview of the Porod analysis with **(a)** evolution of ln [q I(q)] against q^2, overlayed with the points used for the Porod analysis and corresponding fit from **Equation** S11 and **(b)** concentration normalized intensity as a function of q overlayed with the prediction from the Porod fit.

S5. Electron Diffraction Analyses

S5.1 Typical Scanning Nanobeam Electron Diffraction (SNBED) Data

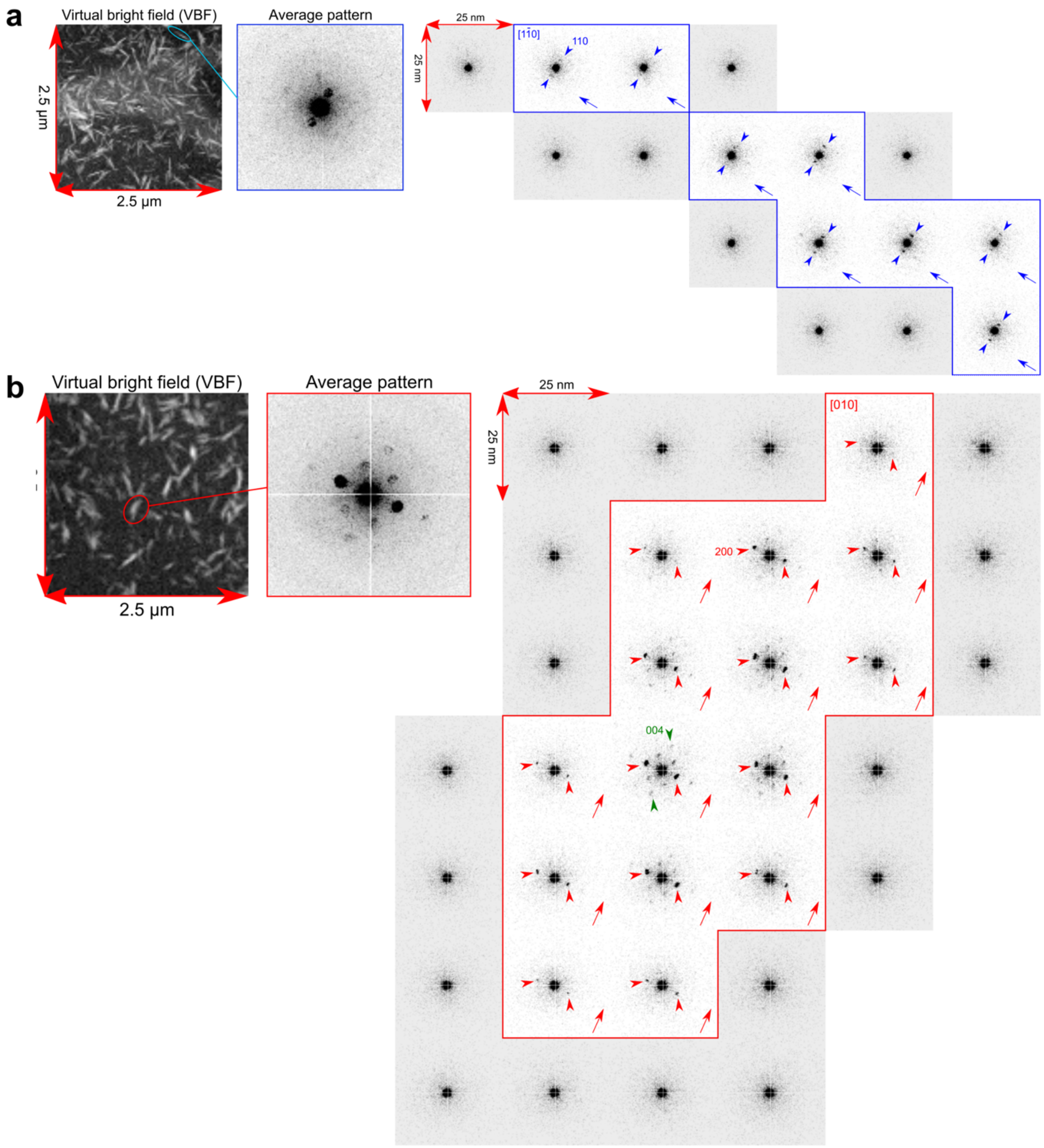

Figure S12. SNBED analysis with virtual bright field image (*left*), average diffraction pattern and ED patterns along a CNC (*right*) for (**a**) a never-centrifuged CNC (**C0**) displaying a [1-10] zone axis and (**b**) a centrifuged CNC (**C3**) displaying a [100] zone axis. The arrows on the corner of each ED pattern indicate the fiber axis.

S5.2 Impact of Favored Interaction Between the Crystallites and the Grid

In a scenario where crystallite association would not be favored along any preferential crystal face, each crystal orientation on the grid would be equiprobable for any population of bundle particles, and as such we would not observe a prevalent orientation. In such a case, even a preferential interaction between the (010) crystal faces and the carbon film on the grid would not account for the observed prevalence of [010] zone-axis orientation. Indeed, upon formation of bundle particles (i.e. from **C0** to **C3**), the (010) faces would be less available (as all the faces would be equally involved in the formation of bundles) resulting in a similar or lower proportion of observed [010] zone axis. Therefore, the sole prevalence of the observation of the [010] zone axis in bundled particles, because of how they lie flat onto the grid, also supports that there is a preferential association of the crystallites through their hydrophobic (100) planes.

S5.3 Selected Area Electron Diffraction (SAED)

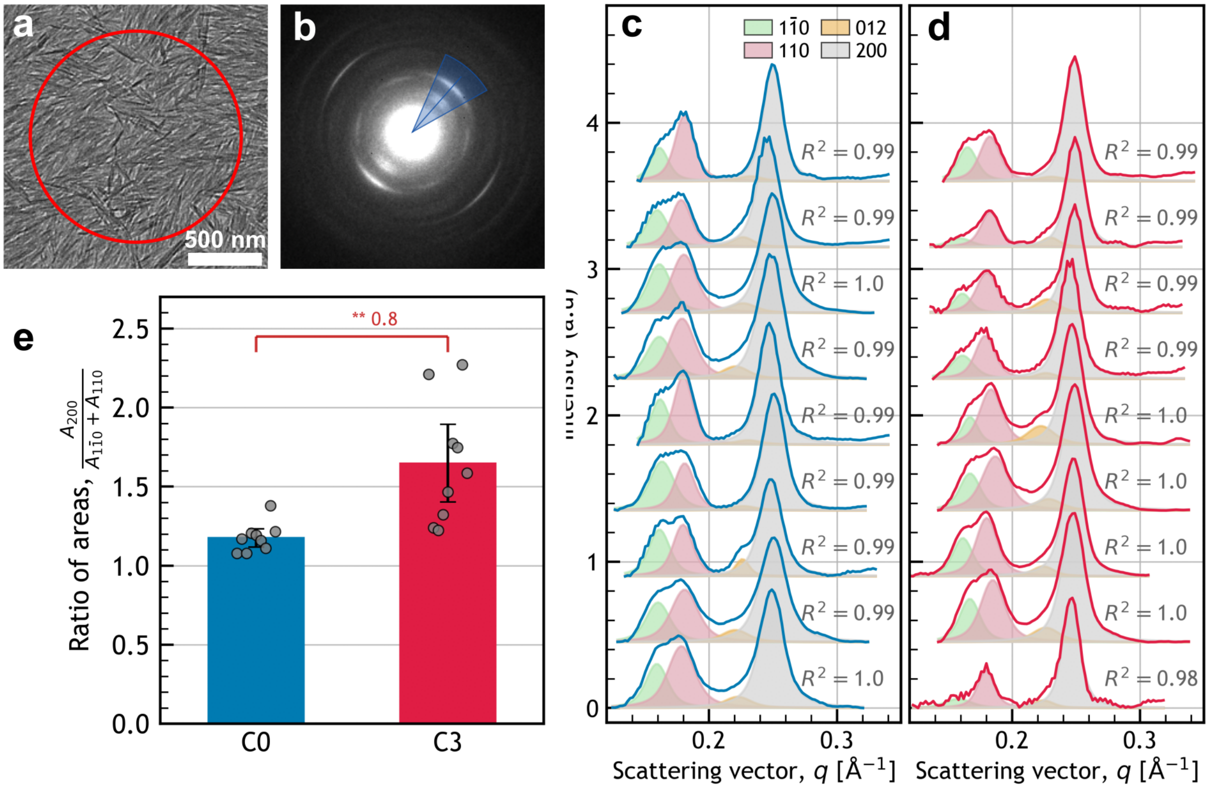

Figure S13. Selected area electron diffraction analysis workflow with **(a)** a typical image and its corresponding area of measurement (*ca* 0.8 µm^2), **(b)** the typical 2D diffraction pattern from a measurement along with the area used to calculate the equatorial profile. **(c-d)** Evolution of the relative intensity against the scattering vector for all the profiles, along with their peak deconvolution, for **(c) C0** and **(d) C3**, and **(e)** the corresponding ratio of the area of the 200 peak over the sum of the area of the 1-10 and 110 peaks, with significance of the difference between the two series estimated through Mann–Whitney U test (p-value < 0.01) and importance of the effect through the absolute point biserial correlation coefficient (r = 0.78).

S6. Interfacial Tension Measurements

S6.1 Extraction of Interfacial Tension from Drop Images

The formation of bundles through the preferential association of crystallites along their hydrophobic faces results to a reduction of the proportion of hydrophobic faces, which could have practical implications for their use as emulsion stabilizer. To investigate if the reduction of the proportion of hydrophobic faces had a significant impact on the CNC amphiphilicity, the interfacial tension between drops of CNC suspensions and hexadecane was measured through pendant drop experiments. The surface charges of the CNCs were screened by NaCl through the preparation of 0.9 wt% CNC suspension in 20 mM NaCl.[9]

To extract the interfacial tension (γ) from the picture of pendant drops, one can rewrite the Young-Laplace equation as:[10]

$$\gamma = \frac{\Delta\rho g D_e^2}{H} \quad \text{(S12)}$$

where $\Delta\rho$ is the density difference between the phases (taken as 224 kg m^{-3}), g is the acceleration of gravity (9.81 m s^{-2}), D_e is the equatorial diameter of the droplet and H is a function of D_s/D_e, with D_s the diameter of the droplet at a height D_e (see example **Figure S14**). The evolution of H as a function of D_s/D_e was previously tabulated, allowing the estimation of γ from D_s/D_e.[11] This approach was implemented in a custom-made Python script to treat the collected images, the software is available on Github under the name DropPyTension.[12] To challenge the accuracy of the script, the surface tension of water in air was calculated from the profile of 20 drops, yielding $\gamma = 71 \pm 4$ mN m^{-1}. This value is close to the accepted value of 72 mN m^{-1} validating the use of this workflow to treat the collected pictures.

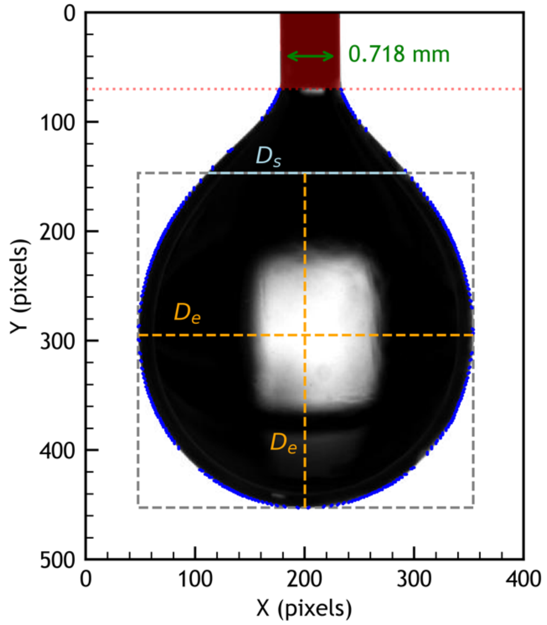

Figure S14. Illustration of pendant drop analysis.

S6.2 Interfacial Tension Measurement of CNC Suspensions

The interfacial tension measurements of the CNC suspensions with hexadecane are presented in **Figure S15**. The γ between water and hexadecane was of 50 ± 3 mN m^{-1}. This is slightly lower than the expected value of 53 mM m^{-1}, which might be attributed to the presence of residual impurities in the hexadecane.[13] The significance of the differences of γ between the samples was estimated through a Student-t test (see **Figure S15** and **Table S12**), but a Mann-Whitney *U* test led to similar conclusions (see **Table S13**). All CNC suspensions, led to a significant lowering of γ, as previously described.[14] The difference of γ between the CNC samples was too small to present any practical relevance but was significant nonetheless, with γ decreasing in the order: **C0-Na**, **C3-Na**, and **C3-Ca**. This small difference is not trivial to relativize and out of the scope of the present work.

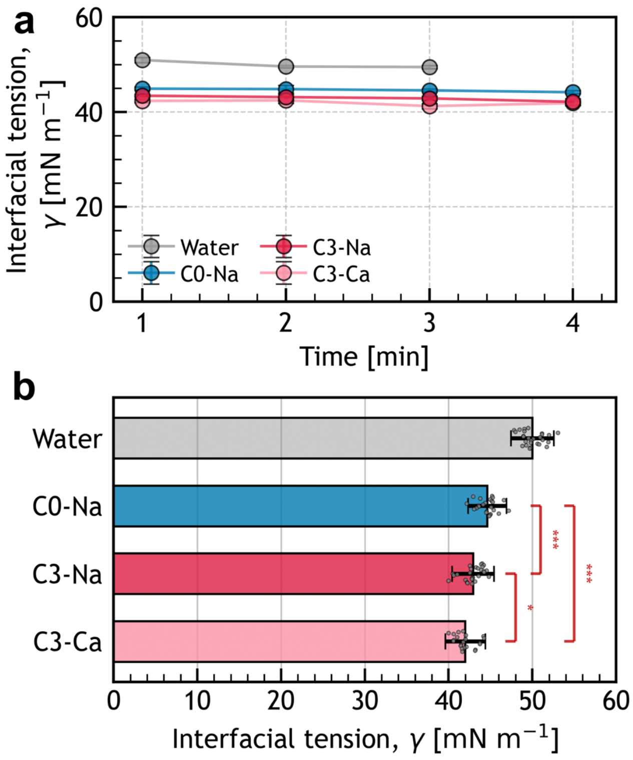

Figure S15. Average interfacial tension (γ) between water or CNC suspension (0.9 wt% CNCs, 20 mM NaCl) drops and hexadecane as a function of time **(a)** and independently of time **(b)**. The distributions were compared pairwise with a Student t-test, statistical significance is indicated by the following *p*-value thresholds: * ($0.05 > p > 0.01$), ** ($0.01 > p > 0.001$), and *** ($p < 0.001$).

Table S12. Absolute Student-t statistics and corresponding *p*-value resulting from the pairwise comparison of interfacial tension values for all samples.

	Water	C0-Na	C3-Na	C3-Ca
**Water**	/	15.5 (0.000)	20.2 (0.000)	22.0 (0.000)
**C0-Na**	15.5 (0.000)	/	4.9 (0.000)	7.5 (0.000)
**C3-Na**	20.2 (0.000)	04.9 (0.000)	/	2.6 (0.013)
**C3-Ca**	22.0 (0.000)	07.5 (0.000)	2.6 (0.013)	/

Table S13. Absolute Mann-Whitney *U* statistics and corresponding *p*-value resulting from the pairwise comparison of interfacial tension values for all samples.

	Water	C0-Na	C3-Na	C3-Ca
**Water**	/	648 (0.000)	729 (0.000)	594 (0.000)
**C0-Na**	648 (0.000)	/	551 (0.000)	494 (0.000)
**C3-Na**	729 (0.000)	551 (0.000)	/	424 (0.011)
**C3-Ca**	594 (0.000)	494 (0.000)	424 (0.011)	/

S7. Capillary images of C3-Ca

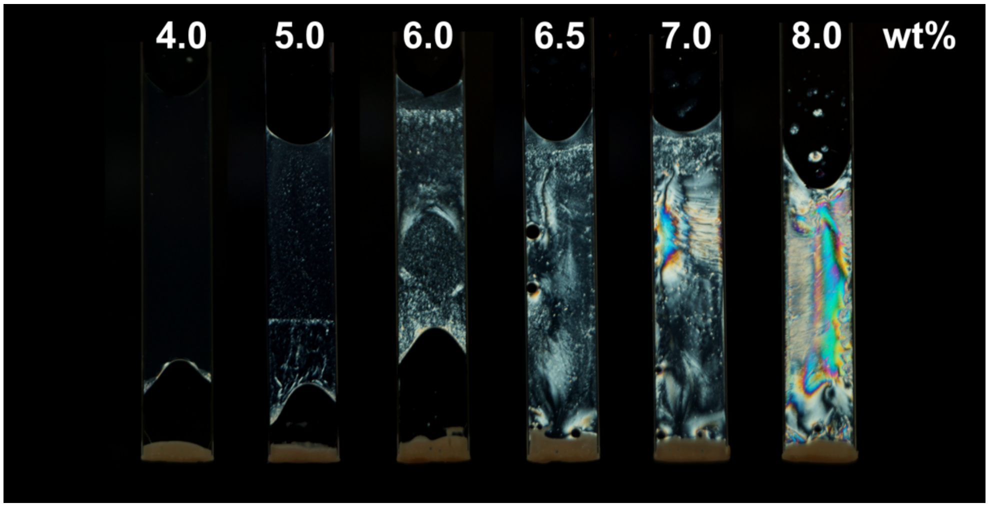

Figure S16. Photograph of a series of capillaries containing CNC suspensions that were exposed to calcium and then dialyzed against ultrapure water (**C3-Ca**) with increasing CNC weight fraction from left to right.

S8 Impact of Salt Condition on CNC Self-Organization

To illustrate the practical significance of our findings for CNC self-organization, we performed a series of experiments using sonicated CNCs (sonication doses 12, 192 and 768 J mL^{-1}) from our previous study.[15] For each sample, a mother suspension of concentrated CNCs (> 10 wt%) was prepared in the presence of NaCl (0.12 mmol g^{-1} of CNC) before being diluted with ultrapure water to obtain a range of CNC volume fractions (Φ) at fixed ratio of NaCl per CNC. The self-assembly behavior of these suspensions was investigated and the Z-average diameter of the CNCs after dilution (0.1 wt%, 1 mM NaCl) was measured and compared to their value before concentration in the presence of salt.

The evolution of the anisotropic volume fraction (φ_{ani}) as a function of Φ for each sample is presented in **Figure S17a**. The φ_{ani} for all samples were superimposed, with an appearance of the anisotropic phase around 2 vol% followed by a sigmoid increase until a fully anisotropic phase is reached around 7 vol%. In **Figure S17b**, the evolution of the corresponding pitches (p) with Φ started to converge whatever the sample sonication history. These results are in stark contrast with the behavior of the same suspensions without salt,[15] for which the self-assembly behavior was dependent of the sonication dose. Moreover, all samples that were concentrated in salt also exhibited an increase of Z-average diameter (**Figure S17c**).

Overall, this experiment shows that a suspension of CNCs reaching 12 mM of NaCl can lead to irreversible morphological changes that impact subsequent self-organization. While adding salt to a CNC suspension is known to shift its biphasic regime to higher Φ and its pitch to lower values,[16] to our knowledge, such superimposition of the self-organization values for different samples was never reported. These observations are coherent with the irreversible formation of bundles upon concentration of the CNCs in the presence of salt, leading to a shift of the biphasic regime toward lower Φ and to lower pitches. However, further work is needed to elucidate the exact origin of the superimpositions of the Φ and pitch values, despite the size differences between the samples.

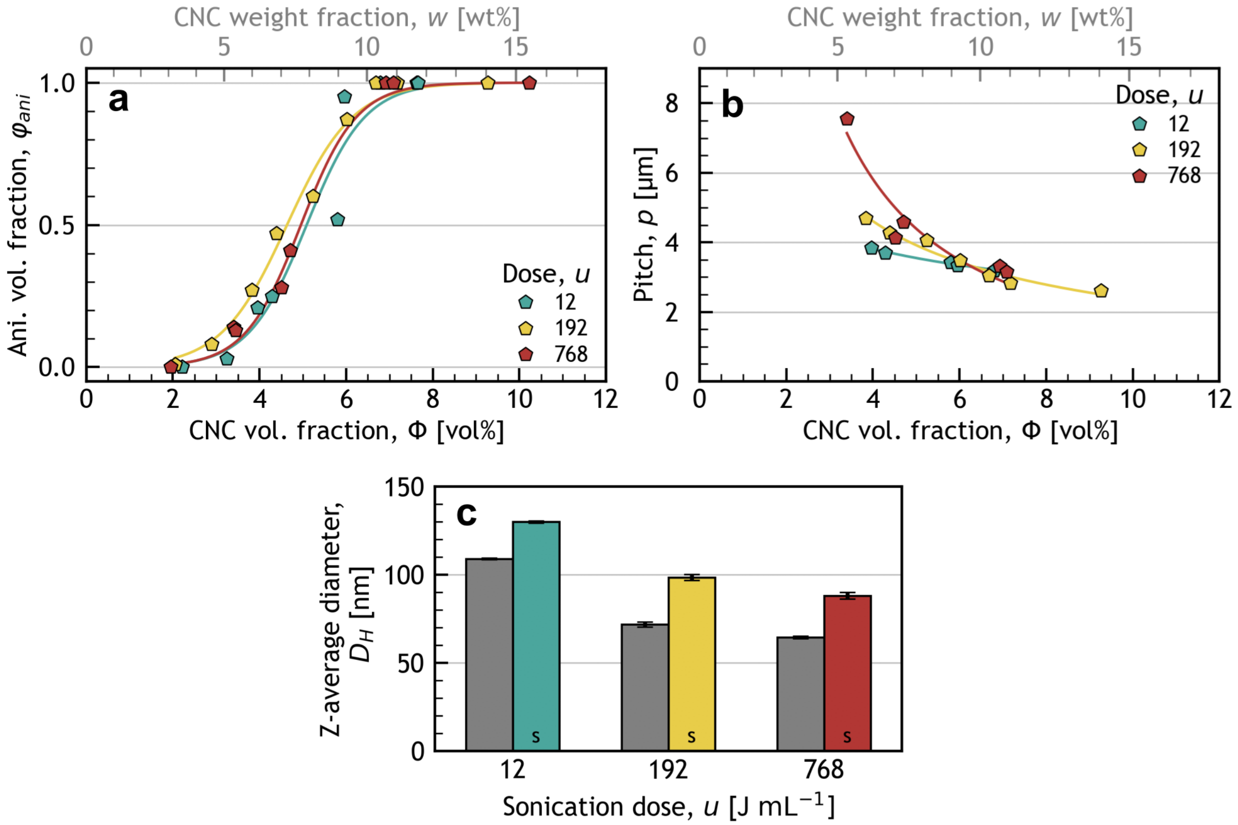

Figure S17. Evolution of the **(a)** anisotropic phase volume fraction (φ_{ani}) and **(b)** pitch (p) as a function of the CNC volume fraction (Φ) for sonicated samples (12, 192 and 768 J mL^{-1}) at fixed ratio of NaCl per CNC (0.12 mmol g^{-1}), after concentration in the presence of NaCl (> 10 wt% CNCs, > 12 mM NaCl). Lines are guides to the eye. **(c)** Z-average diameter (D_H) of the CNCs (at 0.1 wt% in 1 mM NaCl) before (grey) and after (colored, marked with letter 's') concentrating CNC in presence of NaCl salt.

S9. Viscometry

S9.1 Fitting of the Relative Viscosity

In most previous studies, the intrinsic viscosity of CNC suspensions was obtained by fitting the data using a Fedors plot, according to the following expression:[17–23]

$$\frac{1}{2\left(\sqrt{\eta_r}-1\right)}=\frac{1}{[\eta]c}-\frac{1}{[\eta]c_m} \tag{S13}$$

where η_r and $[\eta]$ are respectively the relative and intrinsic viscosities, and c and c_m are respectively the particle concentration and the concentration at maximum packing. However, this approach often yields physically incoherent (i.e. negative or huge) critical concentrations,[24] particularly when applied on low concentrations where small uncertainties lead to huge change of $\sqrt{\eta_r}-1$. Therefore, the authors of the method recommend neglecting data points at low concentrations (*i.e.* below 0.01 g mL^{-1}). In the present study, the viscosity was mainly investigated at low concentration to stay in the dilute regime. Consequently, applying this method to our data still requires to consider low concentrations points (*i.e.* from 0.004 g mL^{-1} to have enough data for a meaningful fit, as illustrated in **Figure S18** and **Table S14**. Instead, we used the Huggins approach, presented in **Equation** 4, which does not suffer from this issue. The intrinsic viscosity values extracted from both methods are quite similar (see **Table S14** and **Table S15**).

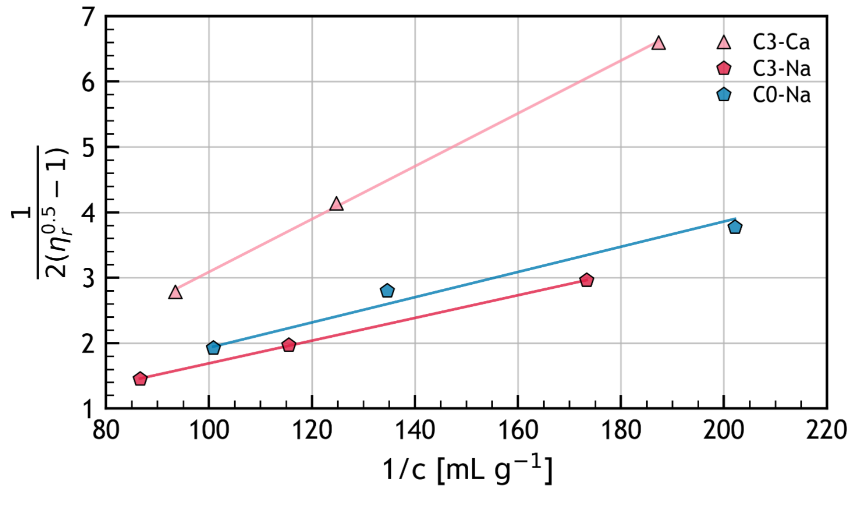

Figure S18. Fedors plot of the relative viscosity data. Values below 0.004 g mL^{-1} (i.e. above than 250 mL g^{-1}) were excluded.

Table S14. Results from the Fedors fit using **Equation** S13, with intrinsic viscosity ([η]), concentration at maximum packing (c_m) and corresponding coefficient of determination (R^2).

Sample	[η] [mL g^{-1}]	c_m [g mL^{-1}]	R^2
**C0**	52 ± 8	6.6E5 ± 9E12	0.967
**C3**	58 ± 1	0.4 ± 0.2	1.000
**C3-Ca**	25 ± 1	4.2E-2 ± 0.5E-2	0.999

Table S15. Results from the Huggins fit using **Equation** 4, with intrinsic viscosity ([η]), Huggins coefficient (k_H) and corresponding coefficient of determination (R^2).

Sample	[η] [mL g^{-1}]	k_H	R^2
**C0**	56 ± 6	0.1 ± 0.2	0.992
**C3**	54 ± 2	0.5 ± 0.1	0.999
**C3-Ca**	25 ± 3	1.6 ± 0.8	0.994

S9.2 Expression for 3D Aspect Ratio

Various expressions for rods and spheroids can be used to relate the 3D aspect ratio (r) to the intrinsic viscosity ($[\eta]$), the Simha relation being the most widespread.[25] However, this expression is specific to rods and the CNC bundles in this work are closer in shape to a spheroid than a cylinder. Therefore, it is more appropriate to use an expression derived from Doi and Edwards, and Brenner for prolate spheroids,[26,27] for which the rotational friction constant is exactly resolved.

The intrinsic viscosity is defined as:

$$[\eta] = \lim_{c \to 0} \frac{1}{c} \frac{\eta - \eta_s}{\eta_s} \tag{S14}$$

where η and η_s are the viscosities of the polymer and of the solvent respectively. In steady shear flow rate for rod-like polymers, the viscosity can be expressed as:[26]

$$\eta = \eta_s + \frac{2}{15} \frac{c}{\rho_p V_p} \zeta_{rot} \tag{S15}$$

where c is the particle concentration, ρ_p is the particle density, V_p is the volume of a particle, and ζ_{rot} is their rotational friction constant. According to Brenner,[27] the rotational friction constant can be expressed as:

$$\zeta_{rot} = 6\eta_s V_p K \tag{S16}$$

where K is a dimensionless scalar coefficient defined in **Equation** S18. Consequently, from **Equation** S14 to S16, the intrinsic viscosity can be simplified as:

$$[\eta] = \frac{4}{5} \frac{K}{\rho_{CNC}} \tag{S17}$$

For spheroids:[27]

$$K = \frac{2(r^2 + 1)}{3(r^2 \alpha_{\parallel} + \alpha_{\perp})} \tag{S18}$$

with:

$$\alpha_{\perp} = \frac{r^2}{r^2 - 1}(1 - \beta) \tag{S19}$$

$$\alpha_{\parallel} = \frac{2}{r^2 - 1}(r^2 \beta - 1) \tag{S20}$$

and where r, the 3D aspect ratio of the spheroid, is expressed as the ratio of its major axis over its minor axis. Finally, for prolate spheroids (i.e. with $r > 1$):

$$\beta = \frac{\cosh^{-1}(\mathrm{r})}{\mathrm{r}\sqrt{\mathrm{r}^2 - 1}} \tag{S21}$$

This yields **Equation** 5, as presented in the Results and Discussion section of the main article.

S10. Fitting the Z-average Diameter as a Function of Ultrasonication Dose

To model the evolution of the hydrodynamic diameter of the CNCs samples with the sonication dose, we propose to use a modified dissociation expression. Thereby, an infinitesimal change of size (*dD_H*) as a function of an infinitesimal change of ultrasonication dose (*du*) is expressed by equation:

$$\frac{dD_H}{du} = -k'(u)\, D_H \tag{S22}$$

with:

$$k'(u) = k^{\alpha}\, \alpha\, u^{\alpha-1} \tag{S23}$$

where k' (mL J^{-1}) is a dose-dependent factor of size decrease, *k* is a constant and α is a stretching exponent. Integrating this expression leads to:

$$D_H(u) = [D_H^0 - D_H^\infty]\exp(-[k\, u]^{\alpha}) + D_H^\infty \tag{S24}$$

where D_H^0 is the size before sonication and D_H^∞ is the size at infinite sonication, fixed at 59 nm for all samples.

To independently isolate the rate of dissociation of the composite particles formed during calcium-induced aggregation, the Z-average diameter of Ca-CNCs ($D_{H,Ca}$) included the fit of the parent sample according to:

$$D_{H,Ca}(u) = \left[\mathrm{D}_{\mathrm{H,Ca}}^0 - D_H^0\right]\exp(-[\mathrm{k}_{\mathrm{Ca}}\, u]^{\alpha_{Ca}}) + D_H(u) \tag{S25}$$

where $D_{H,Ca}^0$ is the size before sonication and $D_H(u)$ is the fitted size of the corresponding parent sample using **Equation** S24. **Equation** S24 and **Equation** S25**,** were used to fit the data presented in **Figure 9** and the corresponding fitting parameters are presented in **Table S16**.

For all samples, the model fitted the data very well for all sonication doses (**Table S16**). Moreover, this approach permits to extract independent factors of size decrease for each type of composite particle, providing information on their relative disassociation behavior. The constants *k* for bundle particles were an order of magnitude smaller than their corresponding k_{Ca} for calcium induced composite particles (**Table S16**). The α coefficients for the bundle particles were also similar and slightly lower than the α_{Ca} for calcium induced composite particles (**Table S16**). Overall, this indicates that sonication energy had a bigger effect on the size of calcium induced composite particles than on that of bundle particles.

Table S16. Best fitting parameters (k or k_{Ca} and α or α_{Ca}) and goodness of fit (R^2 and MSE) for the evolution of the Z-average diameter as a function of the sonication dose using a modified dissociation function (**Equation** S24 and **Equation** S25).

Sample	k or k_{Ca} [mL J^{-1}]	α or α_{Ca}	R^2	MSE
**C0**	0.030 ± 0.003	0.57 ± 0.04	1.00	1.0
**C3**	0.036 ± 0.004	0.53 ± 0.04	0.99	3.5
**C0-Ca**	0.300 ± 0.030	0.66 ± 0.09	1.00	6.4
**C3-Ca**	0.340 ± 0.030	0.74 ± 0.09	1.00	5.2

S11. References

(1) Nobbmann, U. *Derived count rate - what is it?* https://www.malvernpanalytical.com/en/learn/knowledge-center/insights/derived-count-rate-what-is-it (accessed 2025-01-16).

(2) Yap, B. W.; Sim, C. H. Comparisons of Various Types of Normality Tests. *J. Stat. Comput. Simul.* **2011**, *81*, 2141–2155.

(3) Fay, M. P.; Proschan, M. A. Wilcoxon-Mann-Whitney or t-Test? On Assumptions for Hypothesis Tests and Multiple Interpretations of Decision Rules. *Stat. Surv.* **2010**, *4*.

(4) Fritz, C. O.; Morris, P. E.; Richler, J. J. Effect Size Estimates: Current Use, Calculations, and Interpretation. *J. Exp. Psychol. Gen.* **2012**, *141*, 2–18.

(5) *Small Angle X-Ray Scattering*; Glatter, O., Kratky, O., Eds.; Academic Press: London ; New York, 1982.

(6) Feigin, L. A. *Structure Analysis by Small-Angle X-Ray and Neutron Scattering*; Springer: New York, NY, 1987.

(7) Sugiyama, J.; Vuong, R.; Chanzy, H. Electron Diffraction Study on the Two Crystalline Phases Occurring in Native Cellulose from an Algal Cell Wall. *Macromolecules* **1991**, *24*, 4168–4175.

(8) Guinier, A.; Fournet, G. *Small-Angle Scattering of X-Rays*; John Wiley & Sons, Inc: New York, 1955.

(9) Kalashnikova, I.; Bizot, H.; Cathala, B.; Capron, I. Modulation of Cellulose Nanocrystals Amphiphilic Properties to Stabilize Oil/Water Interface. *Biomacromolecules* **2012**, *13*, 267–275.

(10) Andreas, J. M.; Hauser, E. A.; Tucker, W. B. Boundary Tension by Pendant Drops. *J. Phys. Chem.* **1938**, *42*, 1001–1019.

(11) Misak, M. D. Equations for Determining 1/H versus S Values in Computer Calculations of Interfacial Tension by Pendent Drop Method. *J. Colloid Interface Sci.* **1968**, *27*, 141–142.

(12) Ballu, K. *KevinBallu/DropPyTension*. https://github.com/KevinBallu/DropPyTension (accessed 2025-03-05).

(13) Goebel, A.; Lunkenheimer, K. Interfacial Tension of the Water/n-Alkane Interface. *Langmuir* **1997**, *13*, 369–372.

(14) Hu, Z.; Ballinger, S.; Pelton, R.; Cranston, E. D. Surfactant-Enhanced Cellulose Nanocrystal Pickering Emulsions. *J. Colloid Interface Sci.* **2015**, *439*, 139–148.

(15) Parton, T. G.; Parker, R. M.; van de Kerkhof, G. T.; Narkevicius, A.; Haataja, J. S.; Frka-Petesic, B.; Vignolini, S. Chiral Self-Assembly of Cellulose Nanocrystals Is Driven by Crystallite Bundles. *Nat Commun* **2022**, *13*, 2657.

(16) Dong, X. M.; Kimura, T.; Revol, J.-F.; Gray, D. G. Effects of Ionic Strength on the Isotropic-Chiral Nematic Phase Transition of Suspensions of Cellulose Crystallites. *Langmuir* **1996**, *12*, 2076–2082.

(17) Boluk, Y.; Lahiji, R.; Zhao, L.; McDermott, M. T. Suspension Viscosities and Shape Parameter of Cellulose Nanocrystals (CNC). *Colloids Surf. A Physicochem.* **2011**, *377*, 297–303.

(18) Tanaka, R.; Saito, T.; Hondo, H.; Isogai, A. Influence of Flexibility and Dimensions of Nanocelluloses on the Flow Properties of Their Aqueous Dispersions. *Biomacromolecules* **2015**, *16*, 2127–2131.

(19) González-Labrada, E.; Gray, D. G. Viscosity Measurements of Dilute Aqueous Suspensions of Cellulose Nanocrystals Using a Rolling Ball Viscometer. *Cellulose* **2012**, *19*, 1557–1565.
(20) Wu, Q.; Li, X.; Li, Q.; Wang, S.; Luo, Y. Estimation of Aspect Ratio of Cellulose Nanocrystals by Viscosity Measurement: Influence of Aspect Ratio Distribution and Ionic Strength. *Polymers* **2019**, *11*, 781.
(21) Wu, Q.; Li, X.; Fu, S.; Li, Q.; Wang, S. Estimation of Aspect Ratio of Cellulose Nanocrystals by Viscosity Measurement: Influence of Surface Charge Density and NaCl Concentration. *Cellulose* **2017**, *24*, 3255–3264.
(22) Fedors, R. F. An Equation Suitable for Describing the Viscosity of Dilute to Moderately Concentrated Polymer Solutions. *Polymer* **1979**, *20*, 225–228.
(23) Bercea, M.; Navard, P. Shear Dynamics of Aqueous Suspensions of Cellulose Whiskers. *Macromolecules* **2000**, *33*, 6011–6016.
(24) Lenfant, G.; Heuzey, M. C.; van de Ven, T. G. M.; Carreau, P. J. Intrinsic Viscosity of Suspensions of Electrosterically Stabilized Nanocrystals of Cellulose. *Cellulose* **2015**, *22*, 1109–1122.
(25) Li, M.-C.; Wu, Q.; Moon, R. J.; Hubbe, M. A.; Bortner, M. J. Rheological Aspects of Cellulose Nanomaterials: Governing Factors and Emerging Applications. *Adv. Mater.* **2021**, *33*, 2006052.
(26) Doi, M.; Edwards, S. F. *The Theory of Polymer Dynamics*; International series of monographs on physics; Clarendon Press.: Oxford, 1986.
(27) Brenner, H. Rheology of a Dilute Suspension of Axisymmetric Brownian Particles. *Int. J. Multiph. Flow* **1974**, *1*, 195–341.
